# PoroNet: An Intrinsically Interpretable Pore Graph Neural Network for Resolving Pore-Level Adsorption in Metal–Organic Frameworks

**DOI:** 10.1021/acs.jctc.6c00100

**Published:** 2026-05-29

**Authors:** Chao Zheng, Arun Gopalan, Kaihang Shi

**Affiliations:** Department of Chemical and Biological Engineering, 12292 University at Buffalo, The State University of New York, Buffalo, New York 14260, United States

## Abstract

Machine learning (ML) models have been widely used as efficient surrogates to predict adsorption in metal–organic frameworks (MOFs) for gas storage, chemical separations, and catalysis applications. The “black box” nature of these ML models, however, remains a significant barrier between predictions and the design of novel MOFs. In this work, we introduce PoroNet, an intrinsically interpretable graph neural network architecture built on a graph representation of the pore network (i.e., pore graph). In a pore graph, nodes represent individual pores, and edges represent pore connections. PoroNet shows highly accurate predictions of hydrogen (H_2_) uptake and deliverable capacity in MOFs, as well as on a benchmark simulated adsorption data set that contains spherical and linear alkane adsorbates. More importantly, accurate pore-level contributions to total adsorption can be learned using PoroNet either through direct supervised learning or as latent representations while fitting the total adsorption capacity. In direct supervised learning with explicit pore-level labels, we show that the PoroNet architecture is data-efficient, achieving comparable performance to the standard approach with only a fraction of simulation runs needed for model training. The pore-level contribution helps interpret the ML predictions of the total adsorption behavior, identify the key pore properties that govern the adsorption, and provide significant insights into pore engineering. We demonstrate that PoroNet is a powerful tool for high-throughput pore screening and for deriving valuable MOF design rules for cryogenic H_2_ storage applications. Lastly, we discuss the potential of leveraging interpretable ML for scalable scientific and material discovery.

## Graphical abstract

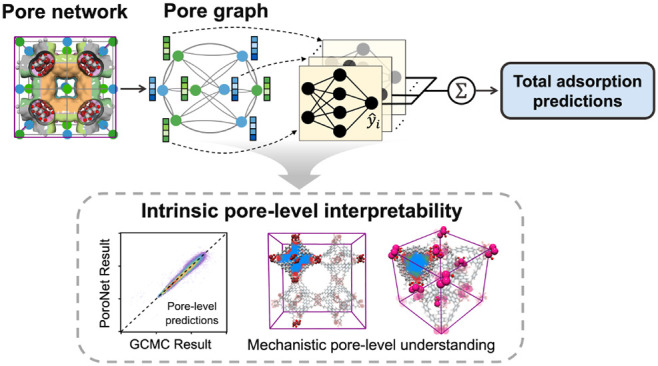

## 1 Introduction

Metal–organic frameworks (MOFs) are a class of crystalline nanoporous materials constructed from metal nodes and organic linkers.^1^ Owing to their ultrahigh surface areas and diverse pore structures, MOFs have emerged as promising candidates for gas storage,^2^ chemical separations,^3^ and catalysis.^4^ The flexibility in choosing metal nodes and organic linkers leads to a seemingly infinite design space for MOFs, presenting both opportunities and significant computational challenges for materials discovery. Grand canonical Monte Carlo (GCMC) simulations have been widely utilized to sift through this large design space by predicting the molecular adsorption properties of MOFs.^5^ However, this approach requires substantial time and computational resources if predictions are needed for thousands or even millions of MOFs. For instance, Bobbitt et al.^6^ spent approximately 500,000 central processing unit (CPU) hours on GCMC simulations to predict the hydrogen (H_2_) storage capacity of 137,953 MOFs.

In recent years, machine learning (ML) has been recognized as a powerful complementary tool to GCMC simulations owing to its higher scalability in predictions.^7^ For example, it was reported that ML models can achieve a speedup of two to three orders of magnitude compared to GCMC simulations for the same task of screening 2,000 MOFs.^8^ While many ML models have achieved high predictive accuracy for gas adsorption, the major challenge lies in their “black-box” nature,^9–14^ with elusive decision-making mechanisms. For the accelerated discovery and design of novel MOFs, understanding how ML models work and uncovering material structure–property relationships from the model’s internal representations are just as important as achieving high predictive accuracy.

Common practices for rationalizing and understanding predictions from “black-box” ML models rely on post-hoc feature importance analyses, such as SHapley Additive exPlanation (SHAP),^15^ permutation importance,^16^ and integrated gradients.^17^ These approaches quantify the contribution of input features to the model outputs, providing indirect explanations for the model’s predictions. For instance, Gurnani et al.^18^ employed the SHAP analysis and revealed that the void fraction, volumetric surface area (VSA), and density are the most important features of MOFs that influence methane (CH_4_) uptake. Similarly, SHAP has been applied to understand ML predictions for carbon dioxide (CO_2_) and nitrogen (N_2_) adsorption,^19,20^ where it identifies the most important features under different thermodynamic conditions. While these post-hoc explainability tools are useful for identifying important features, they attempt to explain ML models after training, rather than building transparency into the model architecture. Through saliency maps, recidivism risk prediction, and model troubleshooting, Rudin^21^ showed that these post-hoc explanation methods may fail to reveal the true decision logic in black-box models. In contrast, an intrinsically interpretable ML model^22^ where the decision-making process is transparent by design, can provide deeper insights into the underlying adsorption mechanisms, deliver predictions with greater confidence, and generate more actionable rules for materials design.

Building an intrinsically interpretable ML model typically involves introducing an inductive bias, i.e., a set of assumptions made by algorithms to give predictions, which essentially defines the reasoning way of a model. For instance, linear regression assumes that the output is a weighted sum of input features. The coefficients explicitly reveal the contribution of each feature to the predictions, making the model intrinsically interpretable. Bucior et al.^23^ utilized the least absolute shrinkage and selection operator (LASSO) regression to predict the deliverable capacity of H_2_ and CH_4_ in MOFs and performed high-throughput screening. By leveraging the natural interpretability of the LASSO model via learned coefficients, they identified optimal adsorption energy ranges for achieving a higher deliverable capacity of H_2_ and CH_4_ in MOFs. Nevertheless, the simplicity of linear models inevitably compromises their capability to learn complex nonlinear correlations that are common in adsorption predictive tasks. A more practical solution is to embed inductive bias into an ML model,^24–31^ enabling partial transparency of the decision-making process while retaining the flexibility of a black-box model. For example, the attention mechanism^32^ has been recently incorporated into various ML architectures, including transformers,^33,34^ feed-forward neural networks,^35^ and graph neural networks,^36,37^ to provide insights into which input features or nodes in a graph the model prioritizes during prediction. It should be noted that the learned attention scores do not always deliver reliable explanations for the model’s behavior because distinct attention distributions could be obtained with the same prediction.^38^ Another notable example is the crystal graph convolutional neural network (CGCNN), which was designed to predict different properties of periodic crystalline materials.^39,40^ In CGCNN, materials are represented by crystal graphs, where nodes and edges in the graph represent atoms and chemical bonds, respectively. The inductive bias lies in the assumption that each node’s features are influenced by its neighbors, and the global material properties can be approximated as an aggregate contribution from all nodes. Such inductive bias enables the CGCNN model to extract contributions from local chemical environments to global properties, such as the atomic contribution to the total formation energy of the material, thus providing interpretability at the model level.^39^ The original formulation of CGCNN, however, is less effective in the case of gas adsorption in porous materials,^14^ as the information on pore space is not explicitly encoded in the model. Petković et al.^41^ attempted to tackle this challenge by explicitly incorporating nodes that represent pores in a zeolite into the crystal graph. Their modified CGCNN model shows accurate predictions for the heat of adsorption and Henry’s coefficients (*K_H_*) for CO_2_ adsorption in zeolites of four topologies. In particular, they introduced an inductive bias in their model that assumes the total heat of adsorption or *K_H_* is a simple sum of contributions from all pore nodes. This partial transparency of the model allowed them to extract qualitative pore-level contributions to the total heat of adsorption from the learned scalar value of each pore node, although whether these learned node values quantitatively correspond to the true pore-level heat of adsorption is still an open question.^41^ While this hybrid crystal graph representation with explicit pore nodes marks a step forward for graph neural networks in terms of adsorption predictions, their definition of pore nodes appears to be limited to zeolites with specific topologies, and the generalizability of the method to other types of porous materials (e.g., MOFs) remains unclear. To date, a critical gap remains in constructing an interpretable ML model with gas-adsorption-aligned inductive bias that rigorously follows physical relations; this gap limits the model’s capability to inform valuable design rules for MOFs with desired gas adsorption properties.

Inspired by the concept of pore graph^42,43^ and pore network modeling^44–46^ from geology, in this work, we propose a novel, intrinsically interpretable graph neural network architecture, PoroNet, to predict gas adsorption in MOFs. PoroNet is built upon a graph representation of the pore network in MOF structures (i.e., pore graph), where nodes and edges in the pore graph represent the individual pores and their connections, respectively. Using published adsorption data sets and H_2_ storage as an application,^47^ we show that the PoroNet architecture can achieve higher predictive accuracy and data efficiency. More importantly, PoroNet offers unprecedented pore-level interpretability via accurate predictions of pore-level adsorption capacity, even without being trained on pore-level labels. Such intrinsic interpretability stems from an inductive bias introduced to the model, reflecting the physical principle: the total adsorption uptake in a MOF is a summation of uptakes across all pores. By leveraging such pore-level interpretability, we are able to perform high-throughput screening for pores in MOFs across the topologically based crystal constructor (ToBaCCo) database^48^ and identify favorable pore types and MOF design rules for cryogenic H_2_ storage applications. Lastly, we benchmark PoroNet against GCMC simulations in terms of pore-level adsorption analysis and discuss the role of interpretable ML in accelerating scientific and material discovery.

## 2 Methods

### 2.1 Selection of MOFs

In this work, we randomly selected 2,000 MOFs from the ToBaCCo 1.0 database,^48^ which contains 13,511 MOFs^49^ with a wide range of framework topologies, pore sizes, void fractions, and surface areas. This ensures that PoroNet is trained and tested on a structurally diverse set of MOFs. We downloaded the MOF structures from the MOFX-DB database^49^ and adopted the same naming method. The list of 2,000 selected MOFs (Selected_MOFs_for_H2_2000.xlsx) can be found at https://github.com/Shi-Research-Group/PoroNet/blob/main/Download_Tobacco_Database.

### 2.2 GCMC Simulations of H_2_ Adsorption

GCMC simulations for H_2_ adsorption in MOFs under cryogenic (77 K/100 bar, 160 K/5 bar)^50^ and ambient conditions (298 K/100 bar, 298 K/5 bar) were performed using the RASPA2 package (version 2.0.47).^51^ The Lennard–Jones (LJ) potential was used to describe the nonbonded interactions with a cutoff radius of 12.8 Å, without tail corrections. Framework atoms remained fixed during the simulations. The LJ parameters for framework atoms were taken from the Universal Force Field (UFF).^52^ We benchmarked two force fields for modeling the H_2_ molecule as a rigid body: the three-site Darkrim-Levesque model^53,54^ with the Feynman–Hibbs (FH) correction and the TraPPE model^55^ without explicit incorporation of the FH correction. GCMC simulations show that the Darkrim–Levesque model leads to better agreement with experimental H_2_ adsorption isotherms in the MOF [see Figure S1 in Supporting Information (SI)], so the Darkrim–Levesque model was used in this study. The Lorentz–Berthelot combining rules were applied to calculate cross-interaction parameters.^56^ No partial charges were assigned to framework atoms. H_2_–H_2_ electrostatics were handled by the Ewald Summation method.^5,57^ The simulation box size was made large enough to ensure that the distance between opposing surfaces in the MOF supercell is at least twice the cutoff radius. The Monte Carlo moves were performed with equal probability; these moves include translation, rotation, reinsertion, insertion, and deletion. The numbers of both initialization and production cycles were set to 3 × 10^3^, which was shown to be enough in previous high-throughput simulations for H_2_.^6,23,54^ Example RASPA2 simulation input files and force field parameters are available in Sec. S1.2.

The number of adsorbed H_2_ molecules in each pore was extracted from GCMC trajectories by post-analyzing the RASPA2 movie file using our in-house Python code (see Sec. S1.3 for details). The GCMC results (in units of molecule number) at both the MOF level and the pore level were converted to density values (g per MOF volume and g per pore volume) and saved for later use in ML training and testing. ML-ready GCMC data are available at https://github.com/Shi-Research-Group/PoroNet/tree/main/GCMC_output.

### 2.3 Pore Graph Neural Network

#### 2.3.1 Generation of Pore Graphs

The overall workflow for PoroNet is shown in Figure 1a. The PoroNet architecture is uniquely enabled by the graph representation of the pore network in the MOF structure, i.e., the pore graph. Figure 1b illustrates the workflow to construct such a pore graph using MOF-5 ^58^ as an example, with more technical details available in Sec. S2.1. First, a Crystallographic Information File (CIF) was imported and converted into an ase.Atom object [Figure 1b­(i)] by the atomic simulation environment (ASE) package (version 3.23.0b1). A dense distance grid, consisting of uniformly distributed points with a spacing of 0.5 Å [Figure 1b­(ii)], was overlaid onto the MOF unit cell to measure the distance from each grid point to the nearest framework atom surface. Grid points that overlap with the framework atoms were labeled as the background (i.e., the space occupied by the framework), and otherwise, as the void space. We found that using a finer grid spacing yields minimal improvement in model predictions while requiring significantly more computational resources (Sec. S2.2). Then, local maxima among the distance grid, subject to predefined filtering criteria (see Sec. S2.1 for details), were detected to serve as seeds for the Watershed segmentation^59^ (Scikit-Image,^60^ version 0.24.0), which subsequently segmented the void space into individual, nonoverlapping pore regions [Figure 1b­(iii)]. In the case of MOF-5, 27 such local maxima were detected, representing 27 individual pores in a single unit cell. By carefully considering the periodic boundary conditions (PBCs; see Table S2), (partial) pores at the unit cell facets, edges, and corners were identified and then grouped to form corresponding complete pores [Figure 1b­(iv)]. All local maxima were then connected based on the detected pore connectivity to form a graph that represents the pore network. This three-dimensional (3D) geometric pore graph contains spatial information about pore arrangement, where nodes in the graph denote individual pores and edges denote pore connections [Figure 1b­(v)]. Finally, the 3D geometric pore graph was simplified to a topological pore graph (simply referred to as “pore graph” in this work) [Figure 1b­(vi)] to preserve connectivity and periodicity information on complete pores.

**Figure 1:**
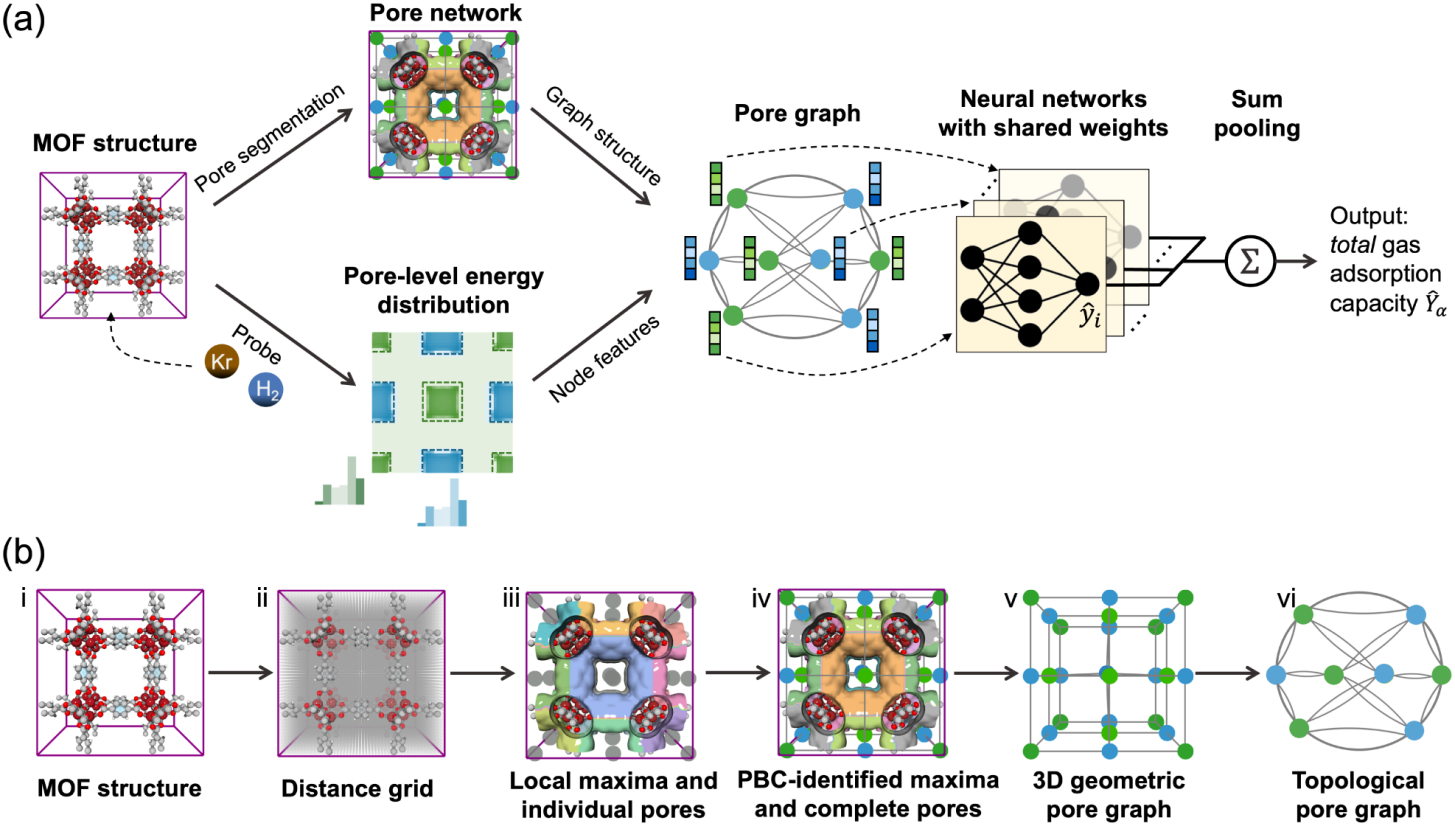
(a) Overall schematics and PoroNet architecture. The pore network (3D geometric pore graph) of MOF *α* is extracted and then converted into the topological pore graph (simply referred to as “pore graph” in this work). Pore-level energy histograms are used as node features in the pore graph. Pore-level features for the node i are then fed into a neural network that shares weights with others to predict the pore-level adsorption capacity, *ŷ*_*i*_. The total adsorption capacity, Y^α , is obtained by summing over all pore-level contributions. (b) Illustration of pore graph construction for MOF-5. (i) The crystal framework structure of MOF-5 is represented as an ase.Atoms object. (ii) The framework structure is overlaid with the distance grid points. (iii) Local maxima in the distance grid are detected, and the void space is segmented into individual pores, with the pore isosurface (1 Å) colored differently for visualizing the pore segmentation. (iv) Segmented (partial) pores at the cell boundary are correctly grouped by considering PBCs, as indicated by the isosurface color. Local maxima are connected when two adjacent pores touch, forming a graph. Individual pores of the same type are represented by the same node color. (v) A 3D geometric pore graph is obtained to represent the pore network in MOF-5. (vi) A topological pore graph can be extracted to preserve connectivity and periodicity information on complete pores in a unit cell.

The pore graph is a highly flexible graph representation of the pore network in a porous structure. It can quantitatively capture global and local pore network and chemistry information through its graph structure and systematic node and edge encoding, similar to how a molecular graph encodes molecular topology and chemistry.^61^ For gas adsorption applications, we extended the MOF-level, one-dimensional (1D) energy histogram features^23^ to their pore-level counterpart and employed pore-level energy histograms as node features to encode both geometric and chemical information on the pore environment (Figure 1a). An energy grid with a resolution of 0.5 Å was used. A probe particle (e.g., center LJ site of H_2_, Kr, Xe, or united-atom methyl group) was placed at each grid point to compute LJ potential energy with all framework atoms, with a cutoff radius of 12.8 Å. Energy grid points belonging to each pore region were then identified, and pore-level energy histograms were calculated. Details on energy histogram parameters are listed in Table S4. In this work, we did not include edge features in the pore graph.

The code to build pore graphs with energy histogram node features from CIFs is available at https://github.com/Shi-Research-Group/PoroNet/tree/main/Pore_Graph_Generation. This specific standalone implementation was adapted from our forthcoming software package, Mofography, which will offer broader accessibility and advanced capabilities for characterizing nanoporous materials using pore graphs.

#### 2.3.2 Architecture of PoroNet

PoroNet is a graph neural network model built on a pore graph, as shown in Figure 1a. It consists of three major components: optional convolutional layers, fully connected layers, and a sum-pooling layer.

Starting from a topological pore graph, the convolutional layers may be used to iteratively update the initial feature vector of node *i* $\left(v_{i}^{\left(0\right)}\right)$ by “convolution” with surrounding pores and pore connections,^39^ as shown in eq 1 for the *t*-th iteration:

1 $$v_{i}^{\left(t\right)}=b+\underset{j\in N\left(i\right)}{\sum }\frac{1}{c_{ji}}v_{i}^{\left(t-1\right)}W$$

where *N*(*i*) is the set of neighbors of node *i*; *c*_*ji*_ is the product of the square root of node degrees (i.e., $c_{ji}=\sqrt{\left|N\left(j\right)\right|}\sqrt{\left|N\left(i\right)\right|}$ ); **b** and **W** are the bias and weight matrix of the convolutional layer, respectively. In preliminary tests, we found that incorporating convolutional layers did not improve the model predictions for the main H_2_ adsorption tasks in this work. This observation is consistent with previous studies that,^8^ at temperatures near or above the adsorbate’s critical temperature, especially for small adsorbates, gas adsorption in MOFs is locally confined, and pore connectivity plays a minor role. Therefore, the current PoroNet implementation consists only of fully connected layers and a sum pooling layer. We expect that convolutional layers may be necessary in the case of capillary condensation (see Section 3.3), where an intricate balance between adsorbate–adsorbate and adsorbate–wall interactions is mediated by the pore network.^8,62^

Consequently, $v_{i}^{\left(0\right)}$ is directly mapped to a scalar, *ŷ*_*i*_, via a fully connected, feed-forward neural network, i.e., a multilayer perceptron (MLP), with *L* hidden layers. Following the common practice in graph neural networks, the same MLP (i.e., identical architecture and shared weights) is applied across all nodes to ensure the model is permutation-invariant to node ordering and enables consistent representation learning.

Then, a sum pooling layer aggregates the node mapping, *ŷ*_*i*_, to predict the total (MOF-level) adsorption property. This sum pooling layer serves as the inductive bias introduced in the PoroNet to guide its learning behavior. Based on different formats of this sum pooling layer, the latent variable *ŷ*_*i*_ is expected to have different physical meanings. When the adsorption density is the target property, the total adsorption for MOF *α* [ Y^α ; e.g., in units of g/L­(MOF)] can be obtained by summing over volumetrically weighted *ŷ*_*i*_ via:

2 $${Ŷ}_{\alpha }=\overset{m}{\underset{i=1}{\sum }}\phi_{i}{ŷ}_{i}$$

where *m* denotes the total number of nodes (pores) in the MOF *α*; *ϕ*_*i*_ = *v*_*i*_/*V*_*α*_ is the volume fraction for pore *i*, where *v*_*i*_ and *V*_*α*_ represent the volumes for pore *i* and MOF *α*, respectively (see Table S5 for the definition of pore volume). In this case, the latent variable *ŷ*_*i*_ represents the pore-level adsorption density [e.g., in units of g/L­(pore)].

On the other hand, if the total number of adsorbed molecules is the property to be predicted, the sum pooling layer becomes

3 $${Ŷ}_{\alpha }=\overset{m}{\underset{i=1}{\sum }}{ŷ}_{i}$$

where *ŷ_i_* now represents the pore-level adsorbed molecule number.

Based on the unique architecture of PoroNet, we introduce two model training strategies. First, PoroNet can be trained on both pore-level labels (*y*_*i*_) and MOF-level labels (*Y*_*α*_), where both labels can be obtained from GCMC simulations (Sec. S1.3). In this case, the loss function incorporates the hierarchical mean absolute errors (MAEs) from both the pore and MOF levels:

4 $$L_{hierarchical}=\frac{\lambda_{1}}{n}\overset{n}{\underset{i=1}{\sum }}\left|{ŷ}_{i}-y_{i}\right|+\frac{\lambda_{2}}{N}\overset{N}{\underset{\alpha =1}{\sum }}\left|{Ŷ}_{\alpha }-Y_{\alpha }\right|$$

where *n* and *N* denote the total number of pores and MOFs in the entire training data set, respectively. Hyperparameters *λ*_1_ and *λ*_2_ were set to 100 and 1, respectively, throughout the work to emphasize the pore-level learning.

Since existing experimental and simulated adsorption databases (e.g., MOFX-DB,^49^ NIST-ISODB^63^) only report total adsorption data, it would be encouraging to train PoroNet with only MOF-level labels and infer pore-level adsorption capacity from the latent representation *ŷ*_*i*_ in the trained model. The loss function in this case is:

5 $$L_{MOF}=\frac{1}{N}\overset{N}{\underset{\alpha =1}{\sum }}\left|{Ŷ}_{\alpha }-Y_{\alpha }\right|$$

To distinguish two training approaches, we refer to the model that is trained on both MOF-level and pore-level labels as PoroNet, and the one that is trained solely on MOF-level labels as PoroNet-Base in the following discussions. More training details, including data splits and optimized hyperparameters, are provided in Sec. S2.5.

After training, PoroNet and PoroNet-Base models were evaluated using the MAEs [eqs 6 and 7] and the coefficients of determination [R^2^: eqs 8 and 9] at both the pore and MOF levels.

6 $$MAE_{pore}=\frac{1}{n}\overset{n}{\underset{i=1}{\sum }}\left|{ŷ}_{i}-y_{i}\right|$$

7 $$MAE_{MOF}=\frac{1}{N}\overset{N}{\underset{\alpha =1}{\sum }}\left|{Ŷ}_{\alpha }-Y_{\alpha }\right|$$

8 $$R_{pore}^{2}=1-\frac{\overset{n}{\underset{i=1}{\sum }}{\left(y_{i}-{ŷ}_{i}\right)}^{2}}{\overset{n}{\underset{i=1}{\sum }}{\left(y_{i}-\mathrm{y̅}\right)}^{2}}$$

9 $$R_{MOF}^{2}=1-\frac{\overset{N}{\underset{\alpha =1}{\sum }}{\left(Y_{\alpha }-{Ŷ}_{\alpha }\right)}^{2}}{\overset{N}{\underset{\alpha =1}{\sum }}{\left(Y_{\alpha }-\mathrm{Y̅}\right)}^{2}}$$

where *y̅* and *Y̅* denote the mean of pore and MOF labels, respectively.

Jupyter Notebooks for building PoroNet and PoroNet-Base are available at https://github.com/Shi-Research-Group/PoroNet/tree/main/PoroNet%26PoroNet-Base.

## 3 Results and Discussion

### 3.1 Joint Learning of Gas Adsorption from Hierarchical Labels

Considering the critical role of H_2_ in the clean energy transition and the fact that few MOFs have significantly surpassed the ultimate system-level storage targets (50 g/L) proposed by the U.S. Department of Energy (DOE),^64^ we mainly apply PoroNet in this work for H_2_ adsorption predictions.

We first examine the PoroNet capability by training it with hierarchical labels that contain both the MOF-level and pore-level labels [eq 4]. Figure 2 presents parity plots comparing GCMC results for H_2_ adsorption density and volumetric deliverable capacity at cryogenic conditions (77 K/100 bar **↔** 160 K/5 bar) against PoroNet predictions on the testing data set (1,000 MOFs and 6,745 pores). Since tiny pores are of less interest in H_2_ storage applications (H_2_ kinetic diameter is 2.89 Å),^65^ we restricted our analysis in this work to pores with a diameter larger than 3 Å (see Table S5 for the definition of pore diameter). As shown in Figure 2a–c, PoroNet demonstrates excellent performance in predicting H_2_ adsorption/deliverable capacity at the MOF level (R^2^: 0.98, MAE: 0.05–0.55 g/L), compared to previous studies.^23^ More importantly, PoroNet exhibits overall good predictions at the pore level (Figure 2d–f, R^2^: 0.92–0.94, MAE: 0.19–1.88 g/L), enabling the quantitatively accurate ML prediction of pore-level adsorption capacity for the first time. While most of the pore-level predictions closely align with exact values from GCMC simulations, some outliers are observed. We found that most outliers come from pores with a pore diameter smaller than 5 Å (Figure S3). This can be attributed to the requirement of higher grid resolution in smaller pores for 1D energy histogram features to work properly. With the current 0.5 Å grid resolution, the energy histograms were found to be dominated by a single positive energy bin, thus becoming less informative as features for accurate ML predictions. Moreover, the coarse resolution of grid points may introduce uncertainty to pore volume estimates for smaller pores, adding potential noise to the converted density labels [g/L­(pore)]. Nonetheless, the total adsorption predictions are less affected by these outliers due to the smaller volumetric weights on these values [see eq 2].

**Figure 2:**
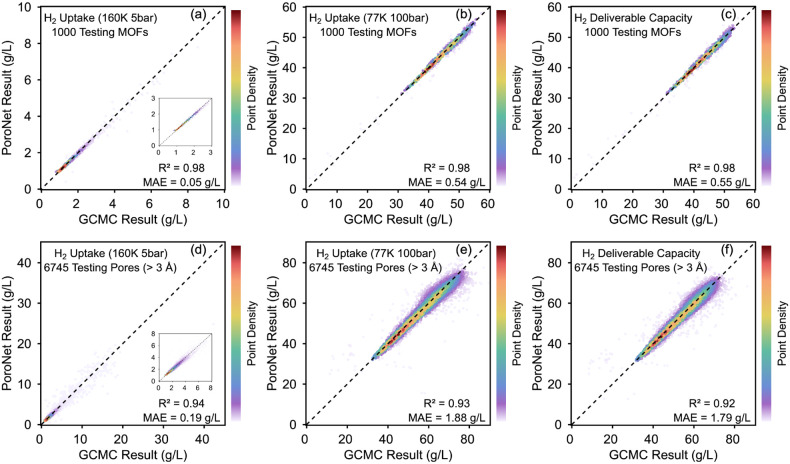
Parity plots comparing GCMC adsorption capacity for H_2_ adsorption (160 K/5 bar and 77 K/100 bar) and deliverable capacity at (a–c) the MOF level and (d–f) the pore level, against PoroNet predictions on the testing set (1,000 MOFs and 6,745 pores). Testing pores are restricted to those with a pore diameter larger than 3 Å; see text for more details. Corresponding parity plots on the training data set are available in Figure S2.

At 160 K/5 bar (Figure 2d), most of the pores are concentrated in the low-loading region (0.8–3.0 g/L), which is favorable for the H_2_ deliverable capacity, as desorption occurs under this condition. As shown in Figure 2e–f, most of the pore-level capacities fall within the range of 32–76 g/L for H_2_ adsorption at 77 K/100 bar and 32–73 g/L for deliverable capacity. The pore-level adsorption density is generally capped at about the bulk liquid density of H_2_ at 77 K (70.89 g/L),^66^ indicating that H_2_ molecules in pores generally do not exhibit excessive “over-packing” beyond the liquid density. In addition, Figure 2f reveals that the maximum H_2_ deliverable capacity of pores (77.73 g/L) exceeds that of MOFs (56.35 g/L, Figure 2c). Considering that the MOF-level deliverable capacity results from a competing effect of pore-level contributions (beneficial) and the volume occupied by the framework atoms (detrimental), this observation suggests strong potential to further enhance MOF-level deliverable capacity through strategic pore engineering. We will return to this topic on rational pore design for high-performing MOFs in Section 3.6. Moreover, we show that PoroNet can accurately predict the volumetric H_2_ adsorption in MOFs under room temperature conditions (298 K/100 bar **↔** 298 K/5 bar), and the testing parity plots are shown in Figure S4.

Beyond the prediction of adsorption density, PoroNet is also capable of predicting the number of adsorbed molecules by incorporating the pore volume as an additional node feature, along with the pore-level energy histogram features. As shown in the testing parity plots in Figure S5, PoroNet demonstrates highly accurate predictions for the number of adsorbed H_2_ molecules, as evidenced by a perfect R^2^ value of 1 and low MAE values. Specifically, PoroNet achieves MAEs of 0.66 and 0.23 at 160 K/5 bar, as well as 11.99 and 4.39 at 77 K/100 bar for the MOF-level and pore-level predictions, respectively. The high accuracy in predicting the molecule number may benefit the application of PoroNet in chemical separation tasks, as the selectivity is more reliably estimated from the number of adsorbed gas molecules^67^ than from the adsorption density, where error propagation is greater in the latter.

### 3.2 Intrinsic Pore-Level Adsorption Prediction from MOF-Level Supervision

Having demonstrated the joint learning capability of the PoroNet model, in this section, we examine the performance and intrinsic interpretability of the PoroNet-Base model that was trained solely on MOF-level loadings using the loss function in eq 5. For MOF-level predictions (Figure 3a–c), PoroNet-Base achieved similar accuracy to that of PoroNet at 160 K (MAE: 0.05 → 0.04 g/L), 77 K (MAE: 0.54 → 0.56 g/L), and for deliverable capacity (MAE: 0.55 → 0.57 g/L). More noticeably, the pore-level adsorption capacity arises as an emergent property of the PoroNet-Base model via the self-learned latent variable *ŷ*_*i*_, even if the PoroNet-Base was not trained explicitly on pore-level adsorption labels. As shown in Figure 3d–f, pore-level predictions from PoroNet-Base show comparable accuracy to that of PoroNet (trained with explicit pore-level labels), with only a slight increase in MAEs at 77 K (MAE: 1.88 → 1.93 g/L) and for deliverable capacity (MAE: 1.79 → 1.83 g/L). Moreover, the high accuracy in pore-level predictions for PoroNet-Base models is also demonstrated with the H_2_ adsorption density at room temperature conditions (Figure S7) and adsorbed molecule numbers at cryogenic conditions (Figure S8). Since existing experimental or computational adsorption databases^49,63^ report only total gas adsorption data, such accurate pore-level predictions achieved via the model’s intrinsic interpretability underscore the practical utility of the PoroNet-Base architecture in scenarios where pore-level labels are not readily available.

**Figure 3:**
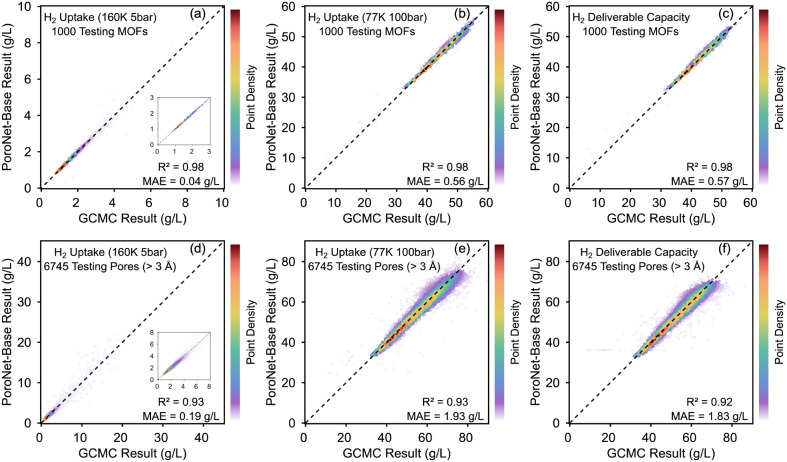
Parity plots comparing GCMC adsorption capacity for H_2_ adsorption (160 K/5 bar and 77 K/100 bar) and deliverable capacity at both (a–c) the MOF level and (d–f) the pore level, against PoroNet-Base predictions on the testing set (1,000 MOFs and 6,745 pores). Testing pores are restricted to those with a pore diameter larger than 3 Å; see text for more details. Corresponding parity plots on the training data are available in Figure S6.

We note that such quantitative pore-level contributions learned by PoroNet-Base represent the core interpretability of the model and are fundamentally different from qualitative interpretability quantities, such as attention scores in prevalent attention-based models. Because the sum pooling layer in the PoroNet architecture explicitly constrains the total adsorption to be the sum of pore-level contributions, the latent variable is directly aligned with a physical quantity, such as pore-level adsorption density or adsorbed molecule count. By contrast, attention scores primarily indicate which parts of the input representation are emphasized by the model in a qualitative manner, and they do not, in general, provide a quantitatively constrained decomposition of total adsorption into pore-wise contributions.

The validation presented here regarding PoroNet-Base’s intrinsic interpretability is purely computational, based on pore-level adsorption quantities extracted from GCMC trajectories. A more rigorous and direct validation would be possible through experimental techniques such as gas adsorption crystallography,^68^ which can provide pore-level adsorption isotherms alongside the total adsorption isotherms. Such an experimental test would help determine whether the learned latent variables *ŷ*_*i*_ indeed correspond to true pore-resolved adsorption capacity in reality. Realizing this experimental validation is beyond the scope of this work but will likely require training PoroNet-Base on experimental total adsorption data, such as those available from NIST-ISODB,^63^ and comparing learned *ŷ*_*i*_ values with experimental pore-level adsorption data for carefully synthesized representative MOFs whose structures closely match the 3D structures used as input to PoroNet-Base.

### 3.3 Benchmark of PoroNet-Base against Baseline ML Models on Existing Adsorption Data Sets

Since PoroNet-Base does not rely on pore-level data for training, we applied PoroNet-Base to published MOF adsorption data sets of various adsorbates (Tables S6 and S8) and benchmarked its performance against baseline ML models.^8,67^ The model benchmark results for the adsorption of Kr, Xe, ethane, and propane are summarized in Table 1, with the corresponding testing parity plots of PoroNet-Base shown in Figure S9. In all adsorption scenarios, PoroNet-Base models outperform the LASSO, random forest (RF), and MLP models that use MOF-level energy histogram features^23^ (i.e., LASSO-EH, RF-EH, and MLP-EH), with a 1.2–62.6% reduction in MAE. The improvement likely arises from the unique architecture of PoroNet-Base, which captures the local adsorption environment within MOFs. The reason that PoroNet-Base significantly outperforms the LASSO-EH in alkane adsorption systems (ethane and propane) is that PoroNet-Base better captures the nonlinear adsorption behavior of the short-chain molecules. PoroNet-Base also achieves higher predictive accuracy in the adsorption of spherical molecules as well as alkanes at low and medium pressures (Ethane-4 bar-298 K, Ethane-20 bar-298 K, Propane-1 bar-298 K, and Propane-5 bar-298 K), compared to baseline ML models with textural features (i.e., RF-textural, RF-textural+K_H_, and MLP-textural). For the adsorption of alkanes at high pressures (Ethane-40 bar-298 K and Propane-10 bar-298 K), PoroNet-Base demonstrates lower accuracy (MAE = 6.65 and 7.21 cm^3^_STP_/cm^3^, respectively) compared to ML models using textural features, such as the MLP-textural model (MAE = 5.60 and 4.50 cm^3^_STP_/cm^3^, respectively). This observation is consistent with known limitations of 1D energy histogram features^67^ and can be attributed to the stronger correlation between the adsorption capacity and structural properties (e.g., surface area and pore diameter) as the pore becomes saturated. In particular, Propane-10 bar-298 K represents an adsorption system where capillary condensation may occur (*T*/*T*_*c*_ = 0.81), and ML predictions were found to be more challenging.^8^ In this case, similar to all other ML models that assume local adsorption and do not explicitly encode the pore connectivity information, the current PoroNet family is expected to be constrained in expressiveness due to its simplified architecture. The performance of the PoroNet-Base model could be improved by implementing graph convolutional layers to explicitly learn the pore network effects and by augmenting node features with additional geometric descriptors.

**Table 1:** Benchmark Comparison of PoroNet-Base with Baseline ML Models on the Testing Data of Various Gas Adsorption Systems^a^

	RF-textural^8^		RF-textural+K_H_ ^8^		MLP-textural^8^		LASSO-EH^67^		RF-EH^67^		MLP-EH (this work)		PoroNet-Base (this work)	
Systems	R^2^	MAE	R^2^	MAE	R^2^	MAE	R^2^	MAE	R^2^	MAE	R^2^	MAE	R^2^	MAE
Kr-1 bar-273 K	0.81	3.20	**0.96**	1.30	N.A.	N.A.	0.85	2.70	0.83	1.90	0.87	1.86	**0.96**	**1.01**
Kr-10 bar-273 K	0.85	10.30	0.94	5.70	N.A.	N.A.	0.96	4.60	0.96	4.60	0.96	3.63	**0.97**	**3.27**
Xe-1 bar-273 K	0.83	9.40	0.92	5.60	N.A.	N.A.	0.95	4.60	0.96	3.40	0.95	4.00	**0.97**	**2.93**
Xe-10 bar-273 K	0.93	13.60	0.97	8.90	N.A.	N.A.	0.96	9.40	0.96	8.10	**0.98**	7.17	**0.98**	**6.24**
Ethane-4 bar-298 K	0.86	12.60	0.96	6.80	0.88	11.90	0.95	7.10	0.96	5.90	**0.98**	4.38	**0.98**	**4.00**
Ethane-20 bar-298 K	0.95	9.80	0.97	7.40	0.94	10.10	0.95	8.90	0.96	7.90	0.97	7.12	**0.98**	**5.68**
Ethane-40 bar-298 K	**0.97**	5.50	**0.97**	**4.90**	0.96	5.60	0.91	8.70	0.92	7.60	0.93	7.88	0.95	6.65
Propane-1 bar-298 K	0.87	11.40	0.94	7.20	0.87	11.40	0.93	8.70	0.96	5.40	**0.98**	4.41	**0.98**	**3.61**
Propane-5 bar-298 K	0.93	9.00	0.95	7.60	0.92	9.40	0.91	13.80	**0.96**	8.30	0.95	8.28	**0.96**	**7.26**
Propane-10 bar-298 K	0.94	4.20	0.94	**4.00**	**0.95**	4.50	0.79	12.40	0.91	7.30	0.80	8.90	0.87	7.21

a RF: random forest, MLP: multilayer perceptron, KH: Henry’s constant, EH: energy histogram. “Textural properties” include the volumetric surface area (VSA), gravimetric surface area (GSA), pore-limiting diameter (PLD), and largest cavity diameter (LCD). Bold numbers indicate the best performance among ML models. The results of the baseline models are taken from previous studies.^8,67^ Units of MAE are in cm^3^_STP_/cm^3^. Corresponding testing parity plots are available in Figure S9.

Overall, PoroNet-Base generally achieves superior predictive accuracy for total adsorption across various adsorbate molecules compared to common baseline models, while maintaining its unique model-level interpretability for pore-level adsorption predictions.

### 3.4 Data Efficiency of PoroNet Architecture

While PoroNet and PoroNet-Base models demonstrate similar predictive accuracy for H_2_ adsorption, as shown in Sections 3.1 and 3.2, PoroNet may exhibit greater data efficiency than PoroNet-Base and other ML models when the size of the training data set is limited. Figure 4 presents the learning curves of LASSO, MLP, PoroNet, and PoroNet-Base models as a function of the number of GCMC-simulated MOFs (ranging from 5 to 300) used for generating the training data at both MOF and pore levels. The models were trained to predict the number of adsorbed H_2_ molecules in MOFs and pores at 160 K/5 bar, and their performance was evaluated on the same testing set as in Sections 3.1 and 3.2. In terms of MOF-level predictions (Figure 4a), PoroNet exhibits a more rapid decline in the testing MAE during the early stage when trained on hierarchical labels derived from 5–10 simulated MOFs, compared to the other ML models that are trained solely on MOF-level labels. Notably, with only 10 simulated MOFs used for training, PoroNet achieves a significantly lower MAE (5.18) than LASSO (9.14), MLP (12.19), and PoroNet-Base (13.42). In addition, PoroNet demonstrates more stable and robust performance, as evidenced by a lower standard deviation of MAE (1.30) compared to the other models (5.52–5.78) across five random splits of training data. Such superior data efficiency and robustness of the PoroNet architecture can be attributed to its unique supervision mode on pore-level data, enabling more granular feature learning compared to conventional approaches. As the number of simulated MOFs used for training increases, the MAE of all models gradually levels off. PoroNet, PoroNet-Base, and MLP reach their performance plateau after ∼200 simulations, whereas LASSO converges much earlier at ∼50 simulations, which is consistent with the literature.^23^ Given 300 simulations for training, PoroNet achieves a low MAE (1.32), comparable to PoroNet-Base (1.18) and MLP (1.33). Although LASSO converges faster, it remains far less accurate with an MAE of 5.14.

**Figure 4:**
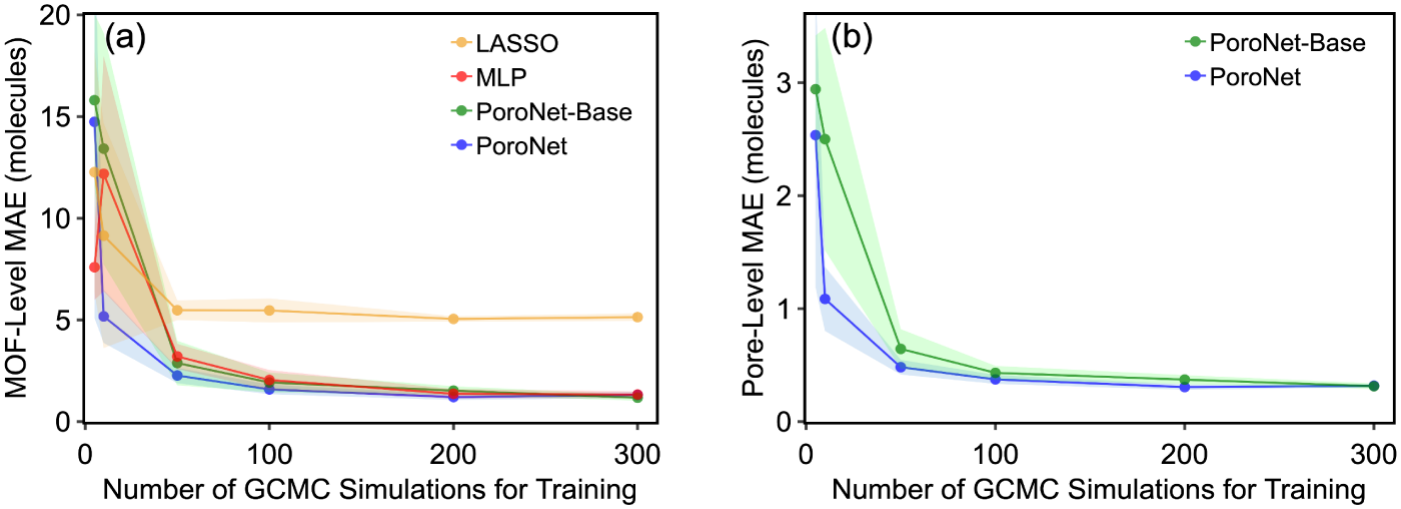
Data efficiency of ML models for predicting the number of adsorbed H_2_ at 160 K/5 bar at the (a) MOF and (b) pore levels. PoroNet and PoroNet-Base use pore-level energy histograms and pore volume as node features, while LASSO and MLP models use the MOF-level energy histogram and MOF cell volume as features. Each point reports an average of testing MAEs from five independent runs with random splits of training samples, and the shaded area represents the corresponding standard deviation. The hyperparameters were reoptimized independently for each of the five runs underlying every point.

In terms of pore-level predictions, similarly, the learning curves in Figure 4b show better data efficiency and robustness of PoroNet compared with PoroNet-Base. With only 10 simulated MOFs for training, PoroNet significantly outperforms PoroNet-Base in both predictive accuracy [average pore-level MAE: 1.09 (PoroNet) versus 2.50 (PoroNet-Base)] and robustness (standard deviation: 0.28 (PoroNet) versus 0.98 (PoroNet-Base)) across different data splits.

The higher data efficiency of PoroNet suggests its practical applicability and reliability in cases where high-quality adsorption data are expensive to generate. For example, although graphical processing unit (GPU)-accelerated software such as gRASPA^69^ has greatly improved the throughput and speed of GCMC simulations using classical force fields, the efficient generation of high-fidelity simulated data using ML force fields^70,71^ is still a challenge. Previous benchmarks^69^ show that GCMC simulation using ML force fields is generally one to two orders of magnitude slower than classical simulation, depending on the choice of ML force field models. Compared to other ML models, PoroNet could achieve the same predictive accuracy with fewer GCMC runs by leveraging pore-level adsorption information from each simulation in addition to common MOF-level data. Likewise, if experiment-level accuracy is desired, PoroNet could be trained on both experimental total and pore-level adsorption data measured from gas adsorption crystallography, effectively reducing the number of expensive gas adsorption experiments.^68^

While PoroNet generally demonstrates superior data efficiency through its dual MOF-level and pore-level training approach, we note that this advantage may not hold consistently across all prediction tasks. For example, in the prediction of cryogenic H_2_ deliverable capacity (density), PoroNet’s performance is comparable to other models (Figure S10). Nevertheless, the model from the PoroNet family (PoroNet or PoroNet-Base) consistently outperforms baseline models in the low-data regime in terms of model accuracy and training robustness.

### 3.5 High-Throughput Screening of MOF Pores for Cryogenic H_2_ Storage Applications

Thanks to the pore-level interpretability of the PoroNet architecture, we are able to perform a systematic analysis of MOF pore environments for cryogenic H_2_ storage applications for the first time—a task that is impractical via conventional analysis of GCMC simulation trajectories. We performed high-throughput screening of pores in ToBaCCo MOFs because of their diverse topologies, varied textural properties, and pore environments.^72^ The ToBaCCo database contains 13,511 MOFs,^48^ of which 13,477 were processed successfully with pore graphs. The pore-level H_2_ deliverable capacities at cryogenic conditions were then predicted using the PoroNet model from Section 3.1 for a total of 89,773 accessible pores (with a diameter larger than 3 Å).

To obtain an initial overview of the entire pore landscape, the Uniform Manifold Approximation and Projection (UMAP),^73^ an unsupervised dimensionality reduction technique, was employed to visualize all pores using the pore-level energy histogram as input features (see Sec. S6.1 for details). As shown in Figure 5a, all pores in ToBaCCo are laid out in a U-shaped structure in the two-dimensional UMAP embedding space, where pores are colored by their predicted pore-level H_2_ deliverable capacity at cryogenic deliverable conditions. Along this U-shaped distribution, the pore-level H_2_ deliverable capacity gradually increases from the low-capacity region at one end (A, blue, with H_2_ deliverable capacity from ∼10 g/L to ∼40 g/L) to the high-capacity region (B, red, with H_2_ deliverable capacity from ∼50 g/L to ∼80 g/L), before decreasing toward the low-capacity region again on the other end of the distribution (C, blue, with H_2_ deliverable capacity of ∼30 g/L). The continuous and smooth transition throughout the whole distribution largely supports the reliability of predictions across the whole database. With a different coloring scheme in terms of pore diameter, Figure 5b shows a monotonically increasing trend in the pore diameter from region A to region C along the data distribution, spanning a wide diameter range from 3 to 79.7 Å. Together with Figure 5a, the results indicate that extremely small or large pores possess low H_2_ deliverable performance.

**Figure 5:**
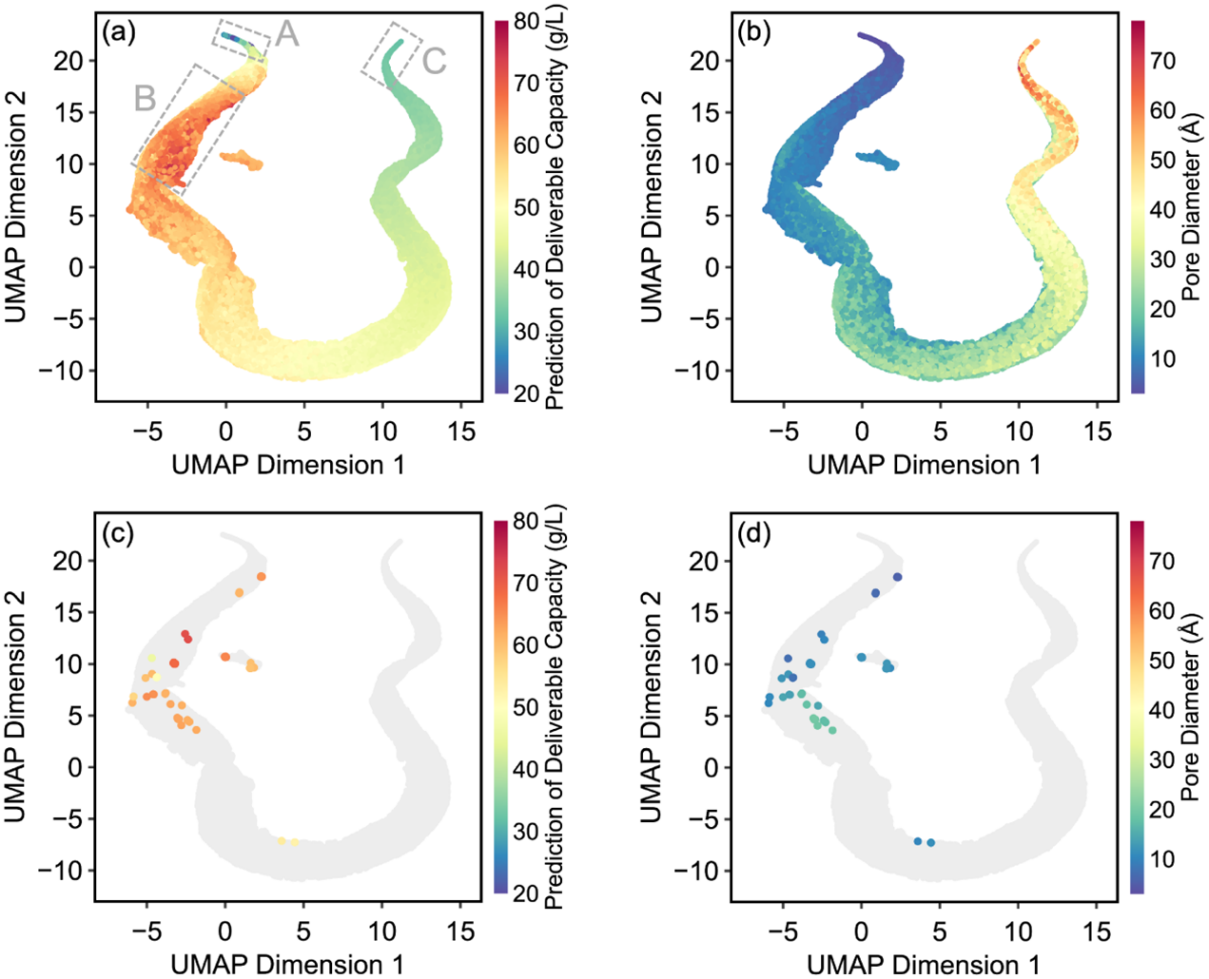
UMAP analysis of 89,773 hypothetical pores from MOFs in the ToBaCCo database and 67 realistic pores from experimental framework materials. Pore-level energy histograms are used as input for dimensionality reduction. Each point represents a pore projected in a two-dimensional UMAP space, with points colored according to the (a, c) predicted pore-level H_2_ deliverable capacity at cryogenic conditions and (b, d) pore diameter. Regions A and C represent two low-capacity regions, and Region B represents the high-capacity region. Realistic pores in the selected framework materials from the literature are highlighted in (c–d) in the same UMAP embedding space; see text for more details.

With the UMAP embedding of hypothetical pores, it is instructive to investigate how realistic pores are positioned in this landscape. The same PoroNet model from Section 3.1 was applied to predict the pore-level H_2_ deliverable capacity at cryogenic conditions in selected high-performing framework materials from the literature: RP-H101,^50^ MOF-5,^74^ HKUST-1,^75^ PCN-61,^54^ ntt_92,^54^ and MFU-4l.^23^ In Figure 5c–d, 67 realistic pores are projected onto the same UMAP embedding space as hypothetical pores in ToBaCCo MOFs. As expected, realistic pores from high-performing framework materials are generally concentrated around the high-capacity region (B) and correspond to medium-range pore sizes in the full spectrum. While no pore-level adsorption data is available in the literature for direct validation, the fidelity of PoroNet predictions and UMAP embedding is indirectly supported by the excellent agreement between the PoroNet-predicted total H_2_ deliverable capacities and the reported simulation/experimental results (Table S9). These results highlight the strong generalizability of PoroNet to unseen framework structures and suggest its practical use in rationalizing the total adsorption performance of real materials based on pore-level contributions.

To obtain a mechanistic insight into high-performing pores, we first take a close look at a strong predictor: the pore diameter. Figure 6 shows the relationship between the cryogenic pore-level H_2_ deliverable capacity and pore diameter. Consistent with the observation from Figure 5a–b, the pore-level H_2_ deliverable capacity initially increases with the pore diameter, reaching a maximum of approximately 78 g/L within the pore size range of 8–11 Å. Beyond 11 Å, the H_2_ deliverable capacity decreases with the pore diameter and gradually converges to a value of ∼32 g/L. To understand the varying H_2_ adsorption behavior across the full pore size range, we visualized three representative pores with small (3.36 Å), medium (10.86 Å), and large (51.53 Å) pore diameters, as shown in the insets a–c in Figure 6. The pore space is color-coded according to the SHAP-identified contributions of energy feature ranges to the cryogenic H_2_ deliverable capacity (Sec. S6.3): red for unfavorable, repulsive regions (>−1 kJ/mol, excluding 0 kJ/mol), blue for favorable, optimally attractive regions (−7 to −1 kJ/mol), and green for unfavorable, strongly attractive regions (≤−7 kJ/mol). Unfavorable regions where the adsorbate-framework energy is 0 kJ/mol are marked separately in orange. For the small pore (Figure 6a), a large portion of the pore space is close to the framework, leading to a high ratio of repulsive regions that hinder the adsorption of H_2_ molecules.^6^ For the large pore (Figure 6c), a large free space is present at the pore center with zero adsorbate-framework interaction energy, and this free space contributes negatively to the H_2_ (volumetric) deliverable capacity.^6^ In contrast, pores with a diameter in the optimal range of 8 to 11 Å (Figure 6b) exhibit the highest proportion of favorable energies (−7 to −1 kJ/mol), which explains their higher performance. In all cases, the unfavorable, strongly attractive regions (≤−7 kJ/mol) occupy only a tiny fraction of the pore volume (0–1.2%), thus their contributions to pore-level H_2_ deliverable capacity are negligible.

**Figure 6:**
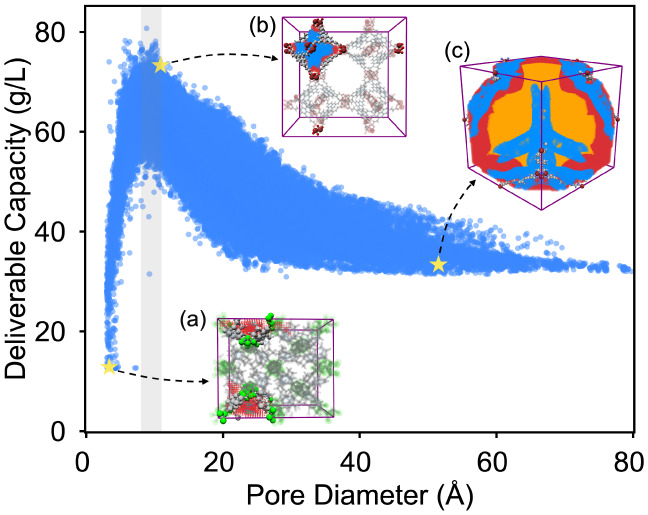
Predicted pore-level H_2_ deliverable capacities at cryogenic conditions by PoroNet as a function of the pore diameter. The data points correspond to the 89,772 accessible pores from MOFs in the ToBaCCo database, with one unphysical pore removed (Figure S13). The gray band indicates the optimal diameter range of 8–11 Å. Representative examples of pores with diameters of (a) 3.36 Å, (b) 10.86 Å, and (c) 51.53 Å are shown as insets, with the pore space colored by energetic favorability: repulsive regions (red), zero-energy regions (orange), favorable regions (blue), and strongly attractive regions (green). See text for details. The corresponding deliverable capacities are 12.83 g/L, 73.70 g/L, and 33.37 g/L, respectively.

Previous studies have reported slightly different optimal pore size ranges for cryogenic H_2_ storage applications. For example, using high-throughput GCMC simulations, Bobbitt et al.^6^ identified the optimal largest cavity diameter (LCD) range of 12–15 Å, although they adopted a slightly different desorption condition (77 K/2 bar). Using the same cryogenic deliverable conditions as this work and a classification ML model, Lu et al.^10^ suggested a broader optimal LCD range of 10–20 Å. We note that these earlier analyses were largely restricted to the MOF-level (global) pore properties, thus obscuring the local adsorption behavior. In contrast, our analysis was performed directly on individual pores via PoroNet’s pore-level interpretability. As a result, the optimal range identified in this work, i.e., 8–11 Å, is more accurate and reflective of pore-level performance.

While the pore diameter is a key factor governing H_2_ adsorption, a large variance in the pore-level H_2_ deliverable capacity is observed for similar pore diameters (Figure 6). For instance, within the optimal diameter range of 8–11 Å, the cryogenic H_2_ deliverable capacity varies from 31.50 to 80.73 g/L, suggesting the influence of additional factors beyond the pore diameter. To explore this, we examined other structural and chemical features, such as pore-level surface area and metal chemistry (see Sec. S6.5). Eventually, we identified pore shape as another important property, one that has been largely overlooked in previous high-throughput screening studies. We characterized the pore shape using the cavity size distribution (Table S5), which measures the distribution of distances between the geometric pore centroid and all other grid points in the pore. A total of 10,953 pores with diameters ranging from 8 to 11 Å were projected into the UMAP embedding space using the cavity size distribution as features. As shown in Figure S11, top-performing pores (cryogenic pore-level H_2_ deliverable capacity >73 g/L) appear to cluster together in the embedding space, suggesting similarities in their pore shapes that favor H_2_ storage. Table S11 lists 121 top-performing pores with visualizations of the pore structure. In this study, we categorize the pore shape primarily based on the spatial arrangement of structural vertices of a pore, where each vertex represents either a metal node or close node pairs in the framework. Apart from the 26 partial pores that were mistakenly identified as complete pores due to limitations of the current pore segmentation algorithm, we found that, among the remaining 95 complete pores, 20 have triangular pyramid shapes, 58 have square bipyramidal shapes, 16 have triangular bipyramidal shapes, and 1 has an irregular shape. This finding suggests that the bipyramidal shape may represent an optimal pore geometry for maximizing the cryogenic H_2_ deliverable capacity.

To further confirm the effect of pore shape, we compared representative pores with similar diameters (10.52–10.88 Å) from realistic and hypothetical high-performing MOFs: MOF-5, HKUST-1, and tobmof-12328. As shown in Table 2, pore 1 from MOF-5 shows a cubic shape and the lowest pore-level H_2_ deliverable capacity (57.17 g/L) among all three pores. Interestingly, the favorable energy region in pore 1 is not cubic in shape but rather bipyramid-like, accounting for 42.6% of the entire pore space. In comparison, pore 2 from HKUST-1 has a higher deliverable capacity (71.79 g/L) than pore 1 and features a truncated square bipyramidal shape, with two opposing vertices removed. The favorable energy region in pore 2 accounts for 53.8% of the pore space, explaining its higher pore-level H_2_ deliverable capacity. Notably, pore 3 from tobmof-12328 exhibits the highest deliverable capacity (73.30 g/L) and the largest ratio of the favorable energy region (56.7%) among the three pores, which may be attributed to the structural advantage of the bipyramidal shape.

**Table 2:**
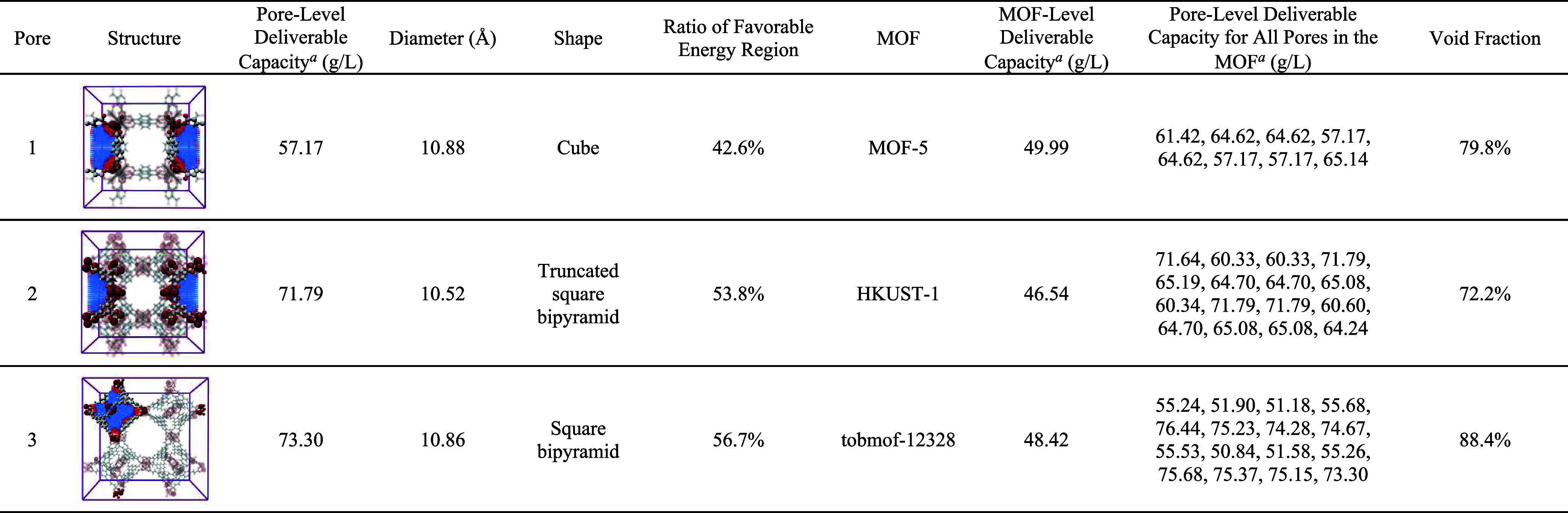
Pore Information and Visualization for Representative MOFs: MOF-5, HKUST-1, and tobmof-12328^a^

a The color scheme of the highlighted pore space is the same as that in Figure 6. H_2_ deliverable capacities in this table are predicted by PoroNet.

### 3.6 Design Insights into High-Performing MOFs

With the optimal pore features identified, the next step is to understand how the superior performance of individual pores can be translated into general design rules for high-performing MOFs. To this end, we examined the correlation between the pore-level and MOF-level H_2_ deliverable capacities. As illustrated in Figure 7, cryogenic pore-level H_2_ deliverable capacity is generally in a positive correlation with the MOF-level deliverable capacity, indicating the critical role that high-performing pores play in determining the overall high MOF performance. Nevertheless, it should be noted that high-performing pores and high-performing MOFs are not causally related, as exceptional pores can exist in mediocre MOFs, and exceptional MOFs can contain mediocre pores. This apparent disconnect can be rationalized by eq 2, which shows that the overall adsorption capacity arises from the weighted contributions of individual pores, where each pore’s adsorption capacity is scaled by its volume fraction that reflects its effective contribution to the MOF-level performance.

**Figure 7:**
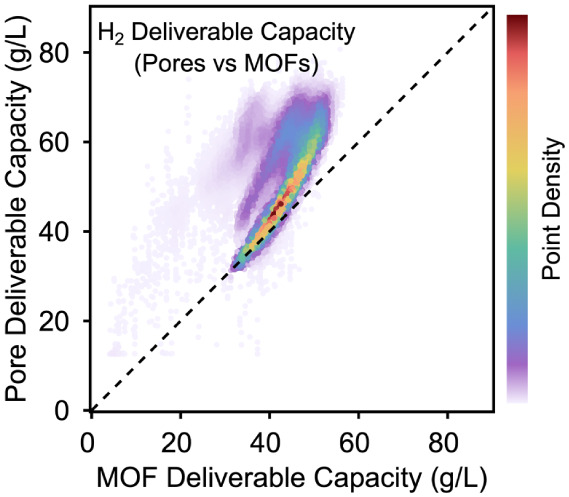
Correlation of pore-level H_2_ cryogenic deliverable capacity with the overall deliverable capacity in the corresponding MOF from the ToBaCCo database. Each point in the plot represents one MOF pore. Both pore-level and MOF-level properties are predicted by PoroNet.

To gain a deeper insight into this noncausal relationship, we extend the analysis from individual pores to their collective effects on MOF performance. As shown in Table 2, MOF-5 has the highest MOF-level H_2_ deliverable capacity (49.99 g/L) compared to HKUST-1 (46.54 g/L) and tobmof-12328 (48.42 g/L), whereas it does not contain the best-performing pores. The higher total deliverable capacity of MOF-5 can be attributed to the combined contribution of the uniform distribution of pore-level deliverable capacities (57.17–65.14 g/L) and a higher void fraction (79.8%), i.e., higher volumetric weights in eq 2. In contrast, while the pore-level deliverable capacities in HKUST-1 shift to a higher range (60.33–71.79 g/L) compared to MOF-5, the void fraction (72.2%) is smaller, resulting in a lower total deliverable capacity. For tobmof-12328, it has the same metal node and topology as HKUST-1, but with larger organic linkers that lead to an expanded framework with the highest void fraction (88.4%). Such expansion increases the diameter of eight octahedral cages (square bipyramid pores) to 10.11–10.86 Å (within the optimal pore diameter range found in Section 3.5) from 5.60 Å for the corresponding cages in HKUST-1, resulting in an exceptional pore-level H_2_ deliverable capacity of 73.30–76.44 g/L. However, the large linkers negatively impact the neighboring pores of octahedral cages in tobmof-12328, shifting their pore size out of the optimal pore size range (8–11 Å). Therefore, the total deliverable capacity of tobmof-12328 is compromised by the formation of these relatively low-performing pores (as low as 50.84 g/L), leading to a higher overall capacity than HKUST-1 but a lower capacity compared to MOF-5. These findings suggest that the design of next-generation MOFs for H_2_ storage should focus not only on incorporating high-performing pores and maximizing the void fraction, but also on ensuring a balanced distribution of pore performance.

### 3.7 From GCMC to Interpretable ML for Scalable Adsorption Analysis

A unique advantage of GCMC simulation is its ability to provide atomistic insights into the adsorption mechanism, such as identifying active sites for adsorption^76^ and elucidating anomalous pore-filling behavior.^62,77^ Due to the significant computational resources required for post-analysis of simulation trajectories, such detailed analysis is usually limited to a handful of structures. In this section, we illustrate how PoroNet-Base can serve as a highly scalable approach for pore-level adsorption analysis at database scale via its intrinsic pore-level interpretability, compared to the conventional approach that requires GCMC simulations and post-analysis of simulation trajectories. Figure 8 illustrates the computational intensity of the conventional approach in extracting pore-level H_2_ adsorption amounts from GCMC trajectories. Assuming there are 90,114 detected pores (regardless of pore size) to be processed across the entire ToBaCCo database, GCMC simulations for 13,511 MOFs at 160 K/5 bar (∼0.3 CPU hours per MOF) and 77 K/100 bar (∼40 CPU hours per MOF) would require a total of ∼544,493 CPU hours. Then, storing 27,022 molecular trajectory files (in PDB format) for both adsorption conditions for post-analysis would require ∼1.5 TB of storage space in total. With approximately 25–30 s for measuring adsorption numbers per pore using our in-house Python code (Sec. S1.3), the estimated time for analyzing GCMC trajectories for all pores across the database would be approximately 1,381 CPU hours.

**Figure 8:**
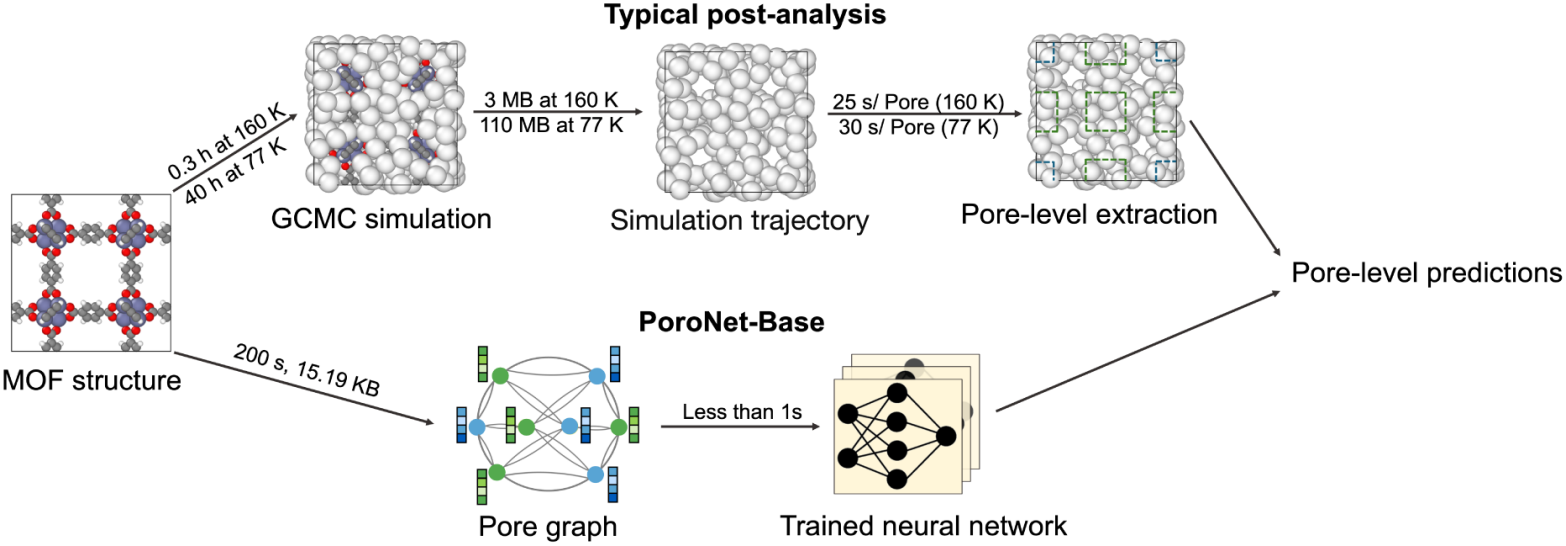
Illustrative comparison of the average computational time and storage requirements for extracting pore-level H_2_ deliverable capacity per MOF, between the typical post-analysis of GCMC simulation trajectories and the PoroNet-Base approach via its intrinsic pore-level interpretability. Details on the computational cost benchmark between the two methods are available in Table S12.

In contrast, the PoroNet-Base model is significantly more efficient and scalable than brute-force post-simulation analysis. As illustrated in Figure 8 and Table S12, the ML workflow only required ∼40,300 CPU hours to generate MOF-level training data for 1,000 ToBaCCo MOFs at both adsorption conditions, 751 CPU hours to generate pore graphs for all 13,511 MOFs, another ∼60 CPU hours to train the models at both adsorption conditions, and less than a minute to predict pore-level H_2_ adsorption capacities for all pores across the database. Therefore, to extract pore-level adsorption capacity for all 13,511 ToBaCCo MOFs (and correspondingly 90,114 pores), the PoroNet-Base approach, with its intrinsic pore-level interpretability, would only require about 41,111 CPU hours and 198 MB of storage, representing 1 and 3–4 orders of magnitude gains in computational and storage efficiency, respectively, compared to conventional trajectory-based analyses (∼545,874 CPU hours and ∼1.5 TB). These scalability and efficiency gains are expected to become even more pronounced when applying the PoroNet approach to larger MOF databases,^78^ which typically contain on the order of 10^5^–10^6^ structures.

## 4 Conclusions

In this work, we have developed PoroNet, an intrinsically interpretable graph neural network model that enables accurate predictions of adsorption in MOFs at both the material and the individual pore levels. PoroNet is built upon the graph representation of the pore network (pore graph) of the MOF structure, where nodes and edges in the graph represent individual pores and their connections, respectively. By encoding the energy histogram of each pore as node features and being trained on the hierarchical labels of both the MOF-level and pore-level H_2_ adsorption data from GCMC simulations, PoroNet exhibits excellent predictive accuracy for cryogenic adsorption properties of H_2_ at both the MOF-level (R^2^: 0.98, MAE: 0.05–0.55 g/L) and the pore-level (R^2^: 0.92–0.94, MAE: 0.19–1.88 g/L). Beyond the adsorption density, we have also shown that PoroNet can accurately predict the number of adsorbed H_2_ molecules in MOFs (R^2^: 1.00, MAE: 0.66–11.99) and pores (R^2^: 1.00, MAE: 0.23–4.39) by incorporating pore volume as an additional node feature. Such high predictive accuracy for molecule number may benefit applications that favor minimal error propagation, such as selectivity predictions for chemical separation applications.

More importantly, even after being trained solely on MOF-level adsorption data, our model (referred to as PoroNet-Base) can still predict pore-level adsorption properties via its learned latent representations, with predictive accuracy (R^2^: 0.98, MAE: 0.04–0.57 g/L at the MOF-level; R^2^: 0.92–0.93, MAE: 0.19–1.93 g/L at the pore level) comparable to that of PoroNet, which is trained explicitly on all-level data. Such intrinsic pore-level interpretability stems from an inductive bias introduced to the model that reflects the physical principle: the total adsorption uptake in a MOF is a summation of uptakes across all pores. Since the training of PoroNet-Base does not require explicit pore-level data, we have benchmarked its performance against common baseline ML models on published adsorption data sets. We have shown that PoroNet-Base generally achieves superior predictive accuracy for the total adsorption across various spherical and short-chain adsorbate molecules, with potential challenges identified for predicting capillary condensation and strong pore saturation. In practice, PoroNet-Base can be readily trained on total adsorption data available in current experimental/simulation adsorption databases, such as MOFX-DB^49^ and NIST-ISODB.^63^ It can then be used to interpret the materials’ adsorption performance via model’s unique pore-level predictions without compromising its predictive accuracy.

In addition, we have benchmarked the data efficiency of the PoroNet family models (PoroNet & PoroNet-Base) against baseline models such as LASSO and MLP. Due to its unique dual MOF-level and pore-level supervision mode, PoroNet enables more granular feature learning compared to conventional approaches, leading to superior data efficiency and training robustness in the extremely low-data regime (5–10 simulated MOFs for training). We note that, while the data advantage of PoroNet may not hold consistently across all prediction tasks, the model from the PoroNet family was found to consistently outperform baseline models in the low-data regime in terms of model accuracy and training robustness. Such high data efficiency of the PoroNet architecture can potentially benefit training cases where high-quality adsorption data are scarce or expensive to generate, such as those obtained from GCMC simulations using the ML force field.

To demonstrate how the intrinsic pore-level interpretability of PoroNet can guide material design, we have applied PoroNet to find the optimal pore types for cryogenic H_2_ storage applications via high-throughput screening of pores from ToBaCCo MOFs. Through systematic analysis of 89,773 hypothetical pores and 67 realistic pores, we identified that pore diameter and shape are two critical factors governing the pore-level cryogenic H_2_ deliverable capacity. Specifically, we found that bipyramid-like pores in the diameter range of 8–11 Å are among the most promising ones for H_2_ storage, owing to their substantially higher volume fraction of favorable energy regions compared to other pore types. To translate these pore-level insights into practical MOF design strategies, we further investigated MOF-5, HKUST-1, and tobmof-12328 and proposed that the overall MOF performance depends not only on individual pore performance but also on the collective contributions from all pores. We suggest that, for cryogenic H_2_ storage applications—and likely many other gas storage applications—the design of next-generation, high-performing MOFs should generally satisfy the following three complementary yet often competing factors: high-performing pores, high void fraction, and balanced distribution of pore-level performance. Although H_2_ adsorption in MOFs has been extensively studied, the optimal pore properties and refined MOF design rules presented in this work demonstrate how ML model’s intrinsic interpretability can shed new light on an old problem.

Using PoroNet-Base as an example, we have illustrated how an interpretable ML model can be used to facilitate the analysis of raw simulation data for adsorption studies. PoroNet-Base was benchmarked against the conventional post-analysis approach for extracting the pore-level H_2_ adsorption capacities, where PoroNet-Base demonstrates superior efficiency in both computational time and storage requirements. This high scalability and intrinsic interpretability make the PoroNet architecture (particularly PoroNet-Base) a powerful tool for mining existing databases and extracting pore-level insights that were previously inaccessible due to computational constraints. Beyond adsorption, the interpretable framework can potentially extend to other important material properties, such as diffusion, providing a promising path for accelerating future scientific and material discoveries.

Finally, we note that the interpretability and predictions of PoroNet depend on the initial pore segmentation, which is inherently nonunique. Nevertheless, the efficient geometric algorithm introduced in this work is highly robust to algorithmic parameters, and the resulting pore segmentation is reproducible and consistent with intuitive, common-sense partitioning. In addition, the current PoroNet architecture does not include graph convolutional layers because the adsorption tasks studied here mainly involve small molecules at temperatures near or above their critical temperatures, where adsorption is largely governed by local environments. Future work will incorporate graph convolution or message-passing layers into PoroNet to explicitly encode pore connectivity. This extension is expected to enable PoroNet to learn more complex behaviors that are strongly coupled with pore networks and chemistry, such as capillary condensation, a fundamental phenomenon relevant to water harvesting, adsorption cooling, and porosity characterization by gas adsorption, where existing models remain limited in predictions.^8^

## Supplementary Material



## Notes

Details on GCMC simulations; pore graph generation; PoroNet architecture, predictions using PoroNet and PoroNet-Base models; data efficiency; high-throughput pore screening; and method benchmarking for extracting pore-level data (PDF)
