# Supplementary material for: PoroNet: An Intrinsically Interpretable Pore Graph Neural Network for Resolving Pore-Level Adsorption in Metal–Organic Frameworks
Source: J Chem Theory Comput. 2026 May 29;22(11):5716–30. doi: 10.1021/acs.jctc.6c00100 (PMC13255179; doi:10.1021/acs.jctc.6c00100)
Supplement: Supplementary file 1 [file ct6c00100_si_001.pdf]

Supporting Information

PoroNet: An Intrinsically Interpretable Pore Graph Neural Network for Resolving Pore-Level Adsorption in Metal-Organic Frameworks

Chao Zheng, Arun Gopalan, and Kaihang Shi*

Department of Chemical and Biological Engineering, University at Buffalo, The State University of New York, Buffalo, New York 14260, United States

**Email: kaihangs@buffalo.edu*

Table of Contents

S1. Grand Canonical Monte Carlo (GCMC) Simulation ... 1
S1.1 Benchmark of Force Fields ... 1
S1.2 Simulation Details and Parameters ... 2
S1.3 Extraction of Pore-Level Labels ... 5
S2. Details on Pore Graphs and PoroNet Architecture ... 5
S2.1 Technical Details on Pore Graph Generation ... 5
S2.2 Effect of Grid Resolution ... 8
S2.3 Details on Energy Histograms ... 9
S2.4 Pore Properties in the Topological Pore Graphs ... 9
S2.5 Training Details ... 10
S3. PoroNet Prediction ... 14
S3.1 Parity Plots on Training Data for H_2 Adsorption under Cryogenic Conditions ... 14
S3.2 Parity Plots on Testing Data Highlighting Small-Pore Outliers ... 15
S3.3 Parity Plots on Testing Data for H_2 Adsorption at Room Temperature ... 16
S3.4 Parity Plots on Testing Data for Predicted Adsorbed H_2 Molecule Numbers ... 17
S4. PoroNet-Base Prediction ... 18
S4.1 Parity Plots on Training Data for H_2 Adsorption under Cryogenic Conditions ... 18
S4.2 Parity Plots on Testing Data for H_2 Adsorption at Room Temperature ... 19
S4.3 Parity Plots on Testing Data for Predicted Adsorbed H_2 Molecule Numbers ... 20
S4.4 Parity Plots on Testing Data for the Prediction of Adsorption of Other Gas Molecules in MOFs ... 21
S5. Data Efficiency of ML Models ... 22
S5.1 Learning Curves for Predicted H_2 Deliverable Capacity ... 22
S6. High-Throughput Screening of Pores ... 23
S6.1 UMAP Analysis ... 23
S6.2 Applying PoroNet to Explain Adsorption in Experimental Nanoporous Materials ... 24
S6.3 SHapley Additive exPlanation (SHAP) Analysis ... 25
S6.4 Visualization of the Significant Outlier ... 27
S6.5 Effects of Pore-Level Surface Area and Chemistry on Cryogenic H_2 Deliverable Capacity ... 28
S6.6 Visualization of Top-Performing Pores ... 29
S7. Methods Benchmark ... 54
References ... 56

S1. Grand Canonical Monte Carlo (GCMC) Simulation

S1.1 Benchmark of Force Fields

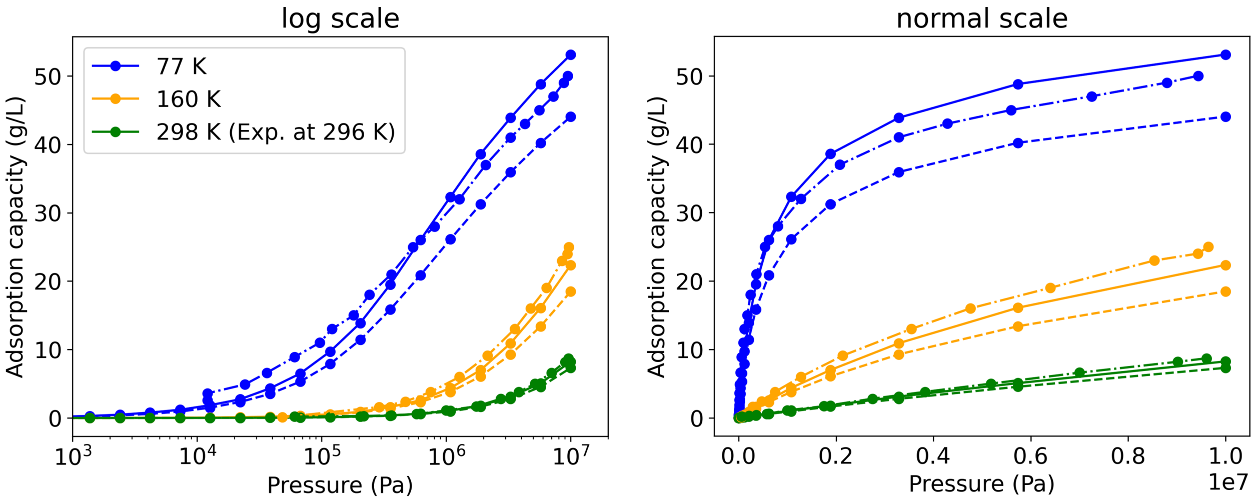

Figure S1. Comparisons of simulated hydrogen (H_2) adsorption isotherms and experimental isotherms in PCN-61 at 77 K (in blue), 160 K (in orange), and 298 K (in green, where experimental data are measured at 296 K). Solid lines represent simulated data using the three-site Darkrim-Levesque model[1, 2]; dashed lines represent simulated data using the TraPPE model[3]; and dash-dotted lines represent experimental data taken from the previous study[2]. Both log (left) and normal (right) scales are shown for the same set of data. Results show that the Darkrim-Levesque model leads to more realistic adsorption isotherms compared to the TraPPE model.

S1.2 Simulation Details and Parameters

Following is a sample input file ("simulation.input") of the GCMC simulations for H_2 adsorption at 160 K/5 bar performed using the RASPA2 package (version 2.0.47)[4]. Samples for other required RASPA2 simulation input files are available at https://github.com/Shi-Research-Group/PoroNet/tree/main/Example_GCMC_input.

```
SimulationType                      MonteCarlo
NumberOfCycles                      3000
NumberOfInitializationCycles        3000
PrintEvery                          1000

Movies                              yes
WriteMoviesEvery                    3

WriteBinaryRestartFileEvery         500
RandomSeed                          10086

ContinueAfterCrash                  no

Forcefield                          local
RemoveAtomNumberCodeFromLabel       yes
CutOffVDW                           12.8

Framework                           0
FrameworkName                       tobmof-2
UnitCells                           3 3 2

ExternalTemperature                 160.0
ExternalPressure                    500000

Component 0 MoleculeName            H2
            MoleculeDefinition      local
            TranslationProbability  0.5
            RotationProbability     0.5
            ReinsertionProbability  0.5
            SwapProbability         1.0
            CreateNumberOfMolecules 0
```

Table S1 Non-bonded 12-6 Lennard-Jones (LJ) potential parameters for GCMC simulations and energy histogram calculations. Parameters for unlike pairs were calculated by the Lorentz-Berthelot combining rules. LJ potential is $v(r) = 4\varepsilon\,[(\sigma/r)^{12} - (\sigma/r)^{6}]$, where r is the distance between any two particles.

Atom Type	ε/k_B (K)	σ (Å)	Atom Type	ε/k_B (K)	σ (Å)
Ac	16.60	3.10	Mo	28.18	2.72
Ag	18.11	2.80	N	34.72	3.26
Al	254.09	4.01	Na	15.09	2.66
Am	7.04	3.01	Ne	21.13	2.66
Ar	93.08	3.45	Nb	29.69	2.82
As	155.47	3.77	Nd	5.03	3.18
At	142.89	4.23	No	5.53	2.89
Au	19.62	2.93	Ni	7.55	2.52
B	90.57	3.64	Np	9.56	3.05
Ba	183.15	3.30	O	30.19	3.12
Be	42.77	2.45	Os	18.62	2.78
Bi	260.63	3.89	P	153.46	3.69
Bk	6.54	2.97	Pa	11.07	3.05
Br	126.29	3.73	Pb	333.59	3.83
C	52.83	3.43	Pd	24.15	2.58
Ca	119.75	3.03	Pm	4.53	3.16
Cd	114.72	2.54	Po	163.52	4.20
Ce	6.54	3.17	Pr	5.03	3.21
Cf	6.54	2.95	Pt	40.25	2.45
Cl	114.21	3.52	Pu	8.05	3.05
Cm	6.54	2.96	Ra	203.27	3.28
Co	7.04	2.56	Rb	20.13	3.67
Cr	7.55	2.69	Re	33.21	2.63
Cu	2.52	3.11	Rh	26.67	2.61
Cs	22.64	4.02	Rn	124.78	4.25
Dy	3.52	3.05	Ru	28.18	2.64

Eu	4.03	3.11	S	137.86	3.59
Er	3.52	3.02	Sb	225.91	3.94
Es	6.04	2.94	Sc	9.56	2.94
F	25.16	3.00	Se	146.42	3.75
Fe	6.54	2.59	Si	202.27	3.83
Fm	6.04	2.93	Sm	4.03	3.14
Fr	25.16	4.37	Sn	285.28	3.91
Ga	208.81	3.90	Sr	118.24	3.24
Ge	190.69	3.81	Ta	40.75	2.82
Gd	4.53	3.00	Tb	3.52	3.07
H	22.14	2.57	Tc	24.15	2.67
He	10.90	2.64	Te	200.25	3.98
Hf	36.23	2.80	Th	13.08	3.03
Hg	193.71	2.41	Ti	8.55	2.83
Ho	3.52	3.04	Tl	342.14	3.87
I	170.57	4.01	Tm	3.02	3.01
In	301.39	3.98	U	11.07	3.02
Ir	36.73	2.53	V	8.05	2.80
K	17.61	3.40	W	33.71	2.73
Kr	166.40	3.64	Xe	221.00	4.10
La	8.55	3.14	Y	36.23	2.98
Li	12.58	2.18	Yb	114.72	2.99
Lu	20.63	3.24	Zn	62.39	2.46
Lr	5.53	2.88	Zr	34.72	2.78
Md	5.53	2.92	H_{h2} [a]	0.0	0.0
Mg	55.85	2.69	H_{com} [b]	36.70	2.96
Mn	6.54	2.64	CH_3_sp^3 [c]	98.00	3.75

[a] H_{h2} refers to the H atoms of the H_2, each carrying a partial charge of +0.468 e. [b] H_{com} refers to the center-of-mass site of H_2, carrying a charge of −0.936 e. For H_2, the Feynman–Hibbs correction was applied to account for quantum effects. [c] CH_3_sp^3 is used for alkane molecules in the calculation of energy histograms.

S1.3 Extraction of Pore-Level Labels

Our Python code enumerated metal-organic frameworks (MOFs) in the GCMC simulation folder and found the molecular trajectories (PDB file). The void space of the MOF was segmented into pore regions, and the periodic boundary conditions (PBCs) were considered to recognize the complete pores (see Section S2.1 for details). Afterwards, the number of gas molecules in each complete pore was counted to get the pore-level adsorption uptake. The Jupyter Notebook for extracting pore-level labels is available at https://github.com/Shi-Research-Group/PoroNet/tree/main/Pore_Labels_Extraction.

S2. Details on Pore Graphs and PoroNet Architecture

S2.1 Technical Details on Pore Graph Generation

To obtain the distance grid of void space, the MOF unit cell is sampled uniformly by placing a point probe along each cell axis at a set spacing (0.5 Å by default). Value at each grid point represents the largest non-overlapping sphere that you can insert at each sample point, while assuming the framework atoms to have sizes specified by their van der Waals radii.[5] This is essentially the shortest distance to the framework surface at each grid point, which by definition, is positive for points in the void space and negative for points in the volume occupied by the framework atoms. This allows us to conceptually split the material unit cell into void space and background, based on a specified "mask thickness" (defaults to 0), which sets the buffer layer distance threshold from the framework atom surface for the split. We found that using a finer grid spacing yields minimal improvement in model predictions while requires significantly more computational resources (Sec. S2.2). Converting the material unit cell to this 3D grayscale image allows powerful image processing algorithms (local maxima detection and regions segmentation) to analyze the pore space easily. Distance maxima (i.e., local maxima) representing pore centers are connected through "windows" (3D constrictions) in the void space. The pore system can appear quite different, such as connected cages or elongated channels, depending on the relative sizes of the maxima, windows, and their connectivity. H-maxima transform algorithm is then used to regularize the local maxima detection to avoid excessive segmentation of void space in the next step. The regularization algorithm only kept local maxima whose distance value (to the nearest framework surface) is higher than the threshold ("threshold_abs" defaults to 1 Å) and the maxima-to-window size difference is at least by "h" (defaults to 0.5 Å). Flood-filling algorithms such as

Classic Watershed can then segment the void space into non-overlapping regions using these filtered local maxima as seeds.[6] The voxels in the background (space occupied by the framework atoms) are assigned the label 0 by the Watershed convention. After getting individual pore regions with corresponding local maxima [Figure 1b (iii)], PBCs should be considered to decide how these regions at the cell boundaries (corners, faces, and edges) should be grouped to obtain complete pores. In MOF-5, we can clearly identify 8 complete pores per unit cell (1/8 th at each corner, 1/4 th at each edge, 1/2 at face centers, 1 at cell center). However, PBCs can get highly nontrivial, especially in systems with non-orthorhombic unit cells and complex pore geometries. Hence, we visually inspected hundreds of materials to derive novel pairwise merging rules involving maxima sizes, locations, and projected overlap, that combine segmented wall regions into "PBC groups". The merging rules for PBC groups are summarized in Table S2. In a simple pore system like MOF-5 (Figure 1b (iv)), eight corners constitute one PBC group, the four edges parallel to each axis form three distinct PBC groups, the opposing face centers form three additional PBC groups, and the pore at the unit cell center gets a group of its own. PBC information can be annotated onto the nodes of the 3D geometric pore graph [Figure 1 (v)], and the graph itself can be abstracted symbolically with fewer nodes, with each node representing one PBC group (or a complete pore in the unit cell), which is called the topological pore graph [Figure 1 (vi)].

Table S2. Rules used in the function “*apply_pbc*” (https://github.com/Shi-Research-Group/PoroNet/blob/main/poronet_functions/compute.py) for merging regions at the boundaries of a 3D segmentation grid.

Step	Rule Description	Implementation Details
1	Identify boundary regions (walls) in each direction (x, y, z)	For each axis, select “wall_cells” number of slices at both the start (left wall) and end (right wall) of the grid.
2	Pair regions on opposite walls	Create pairs of region labels from the left and right walls by flattening and zipping the corresponding slices.
3	Exclude background/zero regions	Only consider pairs where both region labels are nonzero.
4	Compute overlap fraction between paired regions	For each region pair, calculate the fraction of overlap (number of matching voxels) relative to the total voxels of each region on a wall.
5	Apply minimum overlap threshold	Only consider region pairs as candidates for merging if their overlap fraction exceeds minimum_overlap_fraction (default 0.5).
6	Check maxima locations for boundary crossing	For each candidate pair, check if the maxima of the regions are close to the respective walls (within “wall_cells”).
7	Merge based on maxima and radii rules	If both maxima are near their respective walls, merge. If only one is near a wall, merge only if its radius is smaller than the other.
8	Assign merged group labels	Use a graph to track merged regions and assign new group labels to all connected components.

S2.2 Effect of Grid Resolution

We also explored the effect of grids, including the distance grid and energy grid, in the generation of pore graphs and the extraction of pore-level labels. We chose a resolution of 0.25 Å and compared the PoroNet (PoroNet-Base) prediction with that using a resolution of 0.5 Å. Considering the substantial computational resources required when using a grid spacing of 0.25 Å, 350 and 200 MOFs from "Selected_MOFs_for_H2_2000.xlsx" were used here for the training and testing of PoroNet/PoroNet-Base. The comparison of the coefficients of determination (R^2) and the mean absolute errors (MAEs) of H_2 adsorption prediction at 160 K/5bar using different spacing distances on the testing dataset at both the MOF-level and pore-level is shown in Table S3. Using a higher resolution (0.25 Å) yields a minor improvement in predictive accuracy, especially at the pore level, because higher resolution makes a more accurate description of the pore energy distribution. However, it demands computational resources several times greater than those required for a resolution of 0.5 Å when generating pore graphs and extracting pore-level adsorption data. Consequently, the resolution of the distance grid and energy grid was set to be 0.5 Å in this work. In the future, if computational resources allow, higher-resolution grids (e.g., 0.25 Å) could be used to further improve the accuracy of pore-level predictions.

Table S3. Effect of grid resolution on PoroNet and PoroNet-Base prediction of H_2 adsorption at 160 K/5 bar.

Model	Metric	Spacing of 0.5 Å	Spacing of 0.25 Å
PoroNet	MOF-level R^2	0.99	1.00
	MOF-level MAE (g/L)	0.05	0.03
	pore-level R^2	0.89	0.90
	pore-level MAE (g/L)	0.19	0.15
PoroNet-Base	MOF-level R^2	0.99	1.00
	MOF-level MAE (g/L)	0.04	0.03
	pore-level R^2	0.87	0.96
	pore-level MAE (g/L)	0.20	0.16

S2.3 Details on Energy Histograms

Table S4. Optimized parameters of pore-level energy histograms for different probes.[a]

Probe	Range (kJ/mol)	Bin Width (kJ/mol)	Number of Bins
H_{com}	(−10, 0]	1	12
Kr	(−24, 0]	2	14
Xe	(−24, 0]	2	14
CH_3_sp^3	(−24, 0]	2	14

[a] A "strong attractive" bin (E ≤ −10 or −24 kJ/mol) and a "positive" bin (E > 0 kJ/mol) were added to both ends of the energy histogram.

S2.4 Pore Properties in the Topological Pore Graphs

Table S5. Pore properties in the topological pore graphs.

Pore Property	Definition
Diameter	Twice the distance from the local maximum grid point to the nearest framework atom surface
Volume	The product of the MOF unit cell volume and the ratio of number of pore grid points to that of total grid points in the unit cell
Volumetric surface area (VSA)	The surface area of the pore's isosurface at a distance to the framework atom surface equal to the helium radius (1.40 Å), normalized by the volume of the pore
Cavity size distribution	Histogram of distances from all grid points in a pore to the pore geometric centroid

S2.5 Training Details

Training, validation, and test sets split, as well as excluded MOFs, for systems tested in this work, are listed in Table S6.

The neural network was constructed using PyTorch (version 2.2.1, CUDA 11.8),[7] with the Deep Graph Library (DGL, version 2.4.0, CUDA 11.8)[8] employed to process pore graphs. Therefore, the final architecture consists of an input layer, an output layer, and multiple hidden layers with dropout applied to prevent overfitting (the rate was set to a hyperparameter *dropout*). The model was optimized using the Adam optimizer of PyTorch, with L2 regularization (controlled by the coefficient *weight_decay*). A learning rate scheduler (ReduceLROnPlateau) was employed to reduce the learning rate (*lr*) when the validation loss reached convergence, using a reduction factor (*factor*) and a patience step parameter (*patience*). The early stop with the number of patience epochs (*num_patience*) was performed to prevent overfitting (*num_patience* × 0.9 = *patience*), with the maximum number of training epochs set to 5,000. The optimal number and size of hidden layers, corresponding activation functions, as well as other hyperparameters, including *dropout*, *weight_decay*, *lr*, *factor*, and *num_patience*, were selected through hyperparameter optimization using Optuna[9] (version 3.6.1) with 5-fold cross-validation. To ensure reproducibility and consistency, we fixed the random seed of Optuna to 1 (for density prediction) and 10086 (for molecule number prediction), and set 100 trials for the hyperparameter optimization. The searching space and the optimal hyperparameters are listed in Table S7 and Table S8, respectively. Hyperparameters λ_1 and λ_2 in the loss function of PoroNet [Eq. (4)] were chosen as 100 and 1, respectively, to emphasize the pore-level learning.

Table S6. Data splitting for training, validation, and testing, as well as MOFs excluded from datasets in the adsorption of H_2, Kr, Xe, ethane, and propane.

System	Training MOFs	Validation MOFs	Testing MOFs	Excluded MOFs (tobmof-) of type 1[a]	Excluded MOFs (tobmof-) of type 2[b]	Excluded MOFs (tobmof-) of type 3[c]	Excluded MOFs (tobmof-) of type 4[d]
H_2 at160 K/5 bar and 77 K/100 bar (this work)	900	90	1000	4107, 7254, 7496, 4081, 4092, 5740	N.A.	N.A.	10138, 8550, 10330, 1920
H_2 at 298 K/100 bar and 298 K/5 bar (this work)	900	83	1000	4107, 7254, 7496, 4081, 4092, 5740	N.A.	N.A.	10138, 8550, 10330, 1920, 11921, 10132, 12053, 12109, 11969, 11965, 10280
Kr-1bar-273K[10]	900	98	1000	7523	9961	N.A.	N.A.
Kr-10bar-273K[10]	900	94	1000	2170, 6399	N.A.	11177, 5132, 9120, 9952	N.A.
Xe-1bar-273K[10]	900	98	1000	7523	9961	N.A.	N.A.
Xe-10bar-273K[10]	900	95	1000	7715, 2170, 7261, 6399	N.A.	7758	N.A.
Ethane-4bar-298K[10]	900	96	1000	7346, 3345, 7250, 7295	N.A.	N.A.	N.A.
Ethane-20bar-298K[10]	900	95	1000	7254, 4017, 6440, 7346, 4059	N.A.	N.A.	N.A.
Ethane-40bar-298K[10]	900	95	1000	7346, 7250, 3345, 7295, 5185	N.A.	N.A.	N.A.
Propane-1bar-298K[10]	900	97	1000	7261, 6399, 6378	N.A.	N.A.	N.A.
Propane-5bar-298K[10]	900	93	1000	3345, 4017	9961	240, 11437, 4609, 8912	N.A.
Propane-10bar-298K[10]	900	97	1000	7261, 6378	N.A.	2422	N.A.

[a] MOFs whose pore graphs are non-existent due to missing local maxima or are noisy because of a high proportion of smaller pores that need more relaxed parameter sets to build a pore graph. [b] MOFs with grid points strongly overlapping with atoms, leading to numerical error (NaN) in the energy calculation. [c] MOFs whose pore graph generation failed due to excessive memory usage. [d] MOFs that consume excessive memory during the parallel extraction of pore-level labels from GCMC trajectories under high-pressure conditions.

Table S7. Searching space for hyperparameters of the multilayer perceptron.

Hyperparameters	Searching Space
num_patience	10–2000
num_layers	1–3
hidden_size	16–128
hidden_activation (for H_2)	"relu" or "none"
hidden_activation (for Kr, Xe, ethane, and propane)	"relu", "sigmoid", "tanh", "none"
dropout	0.2–0.4
lr	10^{-5}–10^{-1}
weight_decay	10^{-9}–10^{-3}
factor	0.1–0.9

Table S8. Optimized hyperparameters in the multilayer perceptron for different adsorption systems.

Model	Predicted Adsorption System	*num_patience*	*num_layers*	*hidden_size*	*hidden_activation*	*dropout*	*lr*	*weight_decay*	*factor*
PoroNet	H_2-160 K-5 bar-density (g/L)	1070	2	19, 123	none, relu	0.3357	0.0591	3.3559×10^{-8}	0.7972
	H_2-160 K-5 bar-molecule number	492	2	81, 118	relu, relu	0.3205	0.0191	4.5282×10^{-5}	0.3868
	H_2-77 K-100 bar-density (g/L)	1716	2	123, 113	relu, relu	0.2047	0.0587	1.3940×10^{-7}	0.6399
	H_2-77 K-100 bar-molecule number	574	3	121, 26, 79	relu, relu, none	0.2574	0.0709	4.1622×10^{-5}	0.2762
	H_2 deliverable capacity (77 K→160 K)	975	2	101, 110	relu, relu	0.2115	0.0437	1.5377×10^{-8}	0.5690
	H_2-298 K-5 bar-density (g/L)	759	3	80, 119, 74	none, none, relu	0.2446	0.0085	4.2620×10^{-9}	0.4604
	H_2-298 K-100 bar-density (g/L)	1093	3	99, 75, 87	relu, relu, none	0.2083	0.0111	3.8108×10^{-8}	0.1390
	H_2 deliverable capacity (298 K)	1390	2	118, 112	relu, none	0.3782	0.0448	1.7815×10^{-6}	0.8742
PoroNet-Base	H_2-160K-5bar-density (g/L)	532	2	95, 100	relu, none	0.3534	0.0039	5.5008×10^{-7}	0.7845
	H_2-160K-5bar-molecule number	664	3	118, 88, 111	relu, relu, relu	0.2970	0.0070	5.1717×10^{-6}	0.1755
	H_2-77K-100bar-density (g/L)	1016	2	105, 85	relu, relu	0.3492	0.0858	4.9489×10^{-6}	0.4544
	H_2-77K-100bar-molecule number	470	3	109, 28, 99	relu, none, relu	0.2481	0.0695	1.4700×10^{-6}	0.1278
	H_2 deliverable capacity (77 K→160 K)	1099	2	120, 63	relu, relu	0.2455	0.0768	7.1513×10^{-6}	0.6507
	H_2-298K-5bar-density (g/L)	627	1	117	relu	0.3622	0.0015	1.1414×10^{-9}	0.8015
	H_2-298K-100bar-density (g/L)	771	3	94, 63, 21	none, relu, none	0.2168	0.0638	5.4826×10^{-8}	0.7350
	H_2 deliverable capacity (298 K)	1048	3	57, 65, 23	none, relu, none	0.3553	0.0475	3.2864×10^{-6}	0.5509
	Kr-1 bar-273 K-density (cm^3/cm^3)	395	2	91, 102	tanh, relu	0.3882	0.0266	1.0351×10^{-8}	0.8386
	Kr-10 bar-273 K-density (cm^3_{STP}/cm^3)	1138	2	111, 52	relu, relu	0.2075	0.0574	1.2687×10^{-8}	0.6836
	Xe-1 bar-273 K-density (cm^3_{STP}/cm^3)	661	2	86, 119	tanh, relu	0.3187	0.0735	1.5776×10^{-8}	0.6167
	Xe-10 bar-273 K-density (cm^3_{STP}/cm^3)	1125	3	46, 77, 64	relu, tanh, none	0.3571	0.0160	3.2575×10^{-7}	0.6182
	Ethane-4 bar-298 K-density (cm^3_{STP}/cm^3)	584	3	117, 74, 86	tanh, sigmoid, relu	0.3594	0.0217	1.1983×10^{-8}	0.2436
	Ethane-20 bar-298 K-density (cm^3_{STP}/cm^3)	728	3	36, 57, 85	relu, none, tanh	0.2321	0.0078	3.6194×10^{-4}	0.2037
	Ethane-40 bar-298 K-density (cm^3_{STP}/cm^3)	1676	2	66, 96	sigmoid, tanh	0.2838	0.0986	2.5833×10^{-6}	0.8008
	Propane-1 bar-298 K-density (cm^3_{STP}/cm^3)	551	2	115, 81	relu, sigmoid	0.2251	0.0605	1.3029×10^{-7}	0.7194
	Propane-5 bar-298 K-density (cm^3_{STP}/cm^3)	1753	2	124, 126	relu, sigmoid	0.2736	0.0067	1.5029×10^{-6}	0.2075
	Propane-10 bar-298 K-density (cm^3_{STP}/cm^3)	764	2	91, 128	relu, tanh	0.2973	0.0520	2.2364×10^{-4}	0.5907

S3. PoroNet Prediction

S3.1 Parity Plots on Training Data for H_2 Adsorption under Cryogenic Conditions

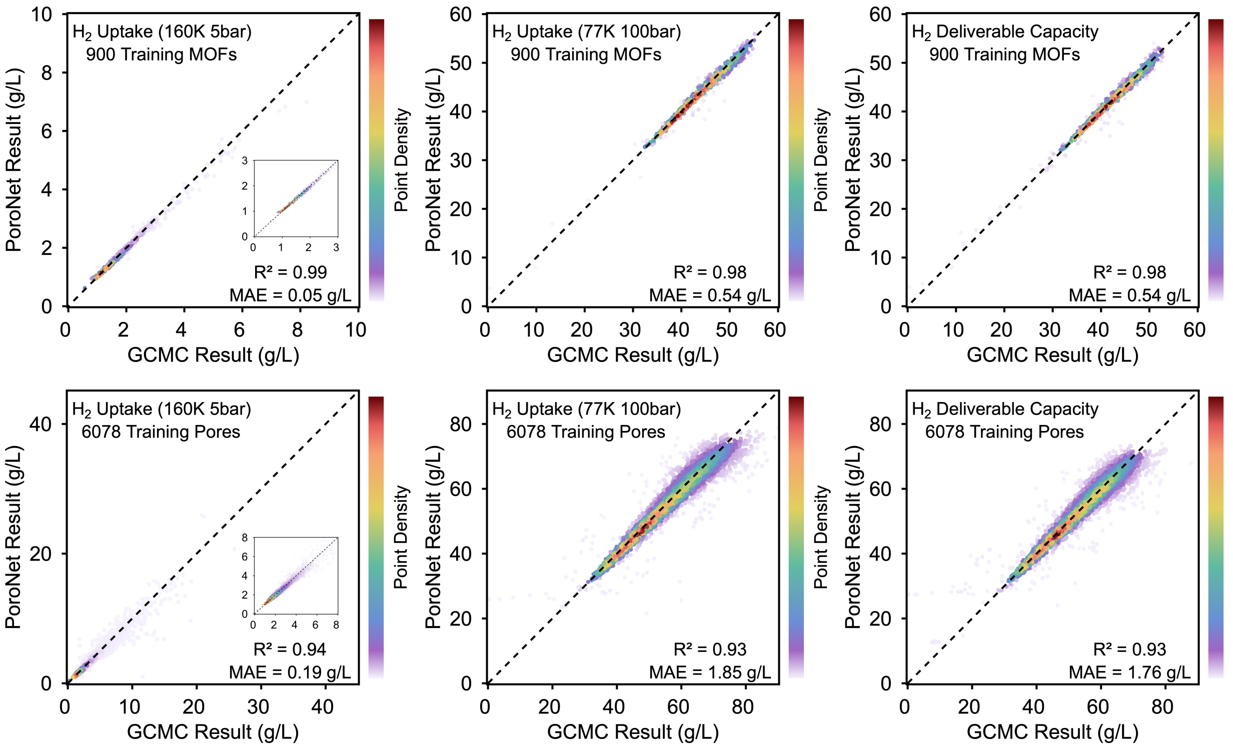

Figure S2. Parity plots comparing GCMC adsorption capacity for H_2 adsorption (160 K/5 bar and 77 K/100 bar) and deliverable capacity at both MOF level and pore level, against PoroNet predictions on the training set (900 training MOFs, 6,078 training pores). PoroNet models were trained on both pore-level and MOF-level labels.

S3.2 Parity Plots on Testing Data Highlighting Small-Pore Outliers

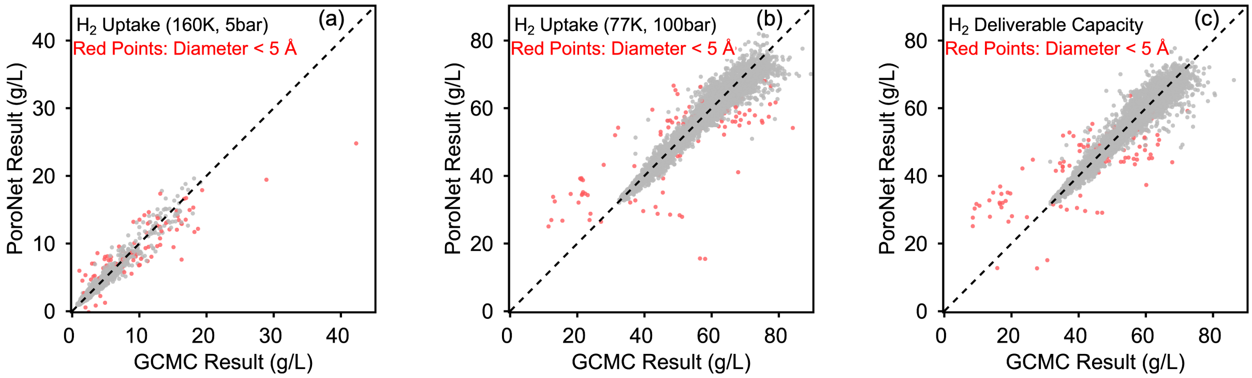

Figure S3. Parity plots comparing GCMC adsorption capacity for H_2 adsorption (160 K/5 bar and 77 K/100 bar) and deliverable capacity at the pore level, against PoroNet predictions on the testing set (6745 testing pores). PoroNet models were trained on both pore-level and MOF-level labels. The data in these plots are the same as those in Figures 2d–f in the main text, but with some outliers highlighted in red. These outliers (red points) represent the pores with a diameter below 5 Å.

S3.3 Parity Plots on Testing Data for H_2 Adsorption at Room Temperature

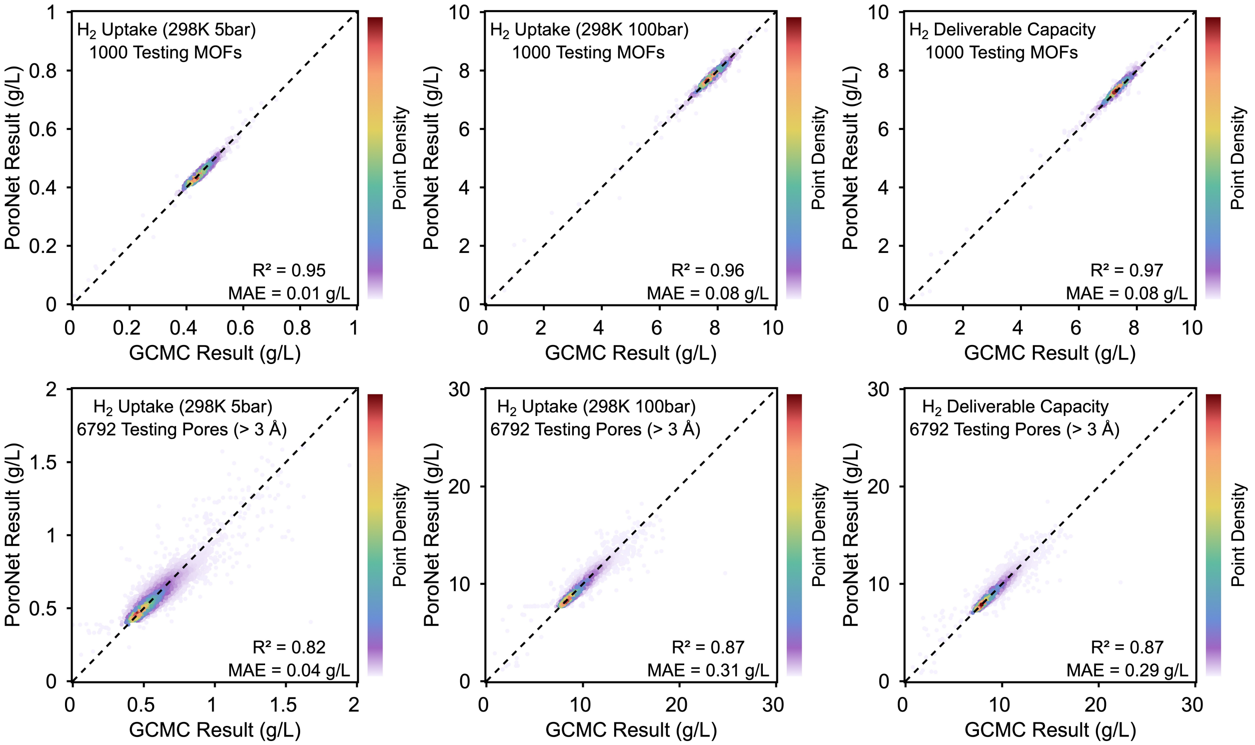

Figure S4. Parity plots comparing GCMC adsorption capacity for H_2 adsorption (298 K/5 bar and 298 K/100 bar) and deliverable capacity at both MOF level and pore level, against PoroNet predictions on the testing set (1,000 testing MOFs, 6,792 testing pores). PoroNet models were trained on both pore-level and MOF-level labels.

S3.4 Parity Plots on Testing Data for Predicted Adsorbed H_2 Molecule Numbers

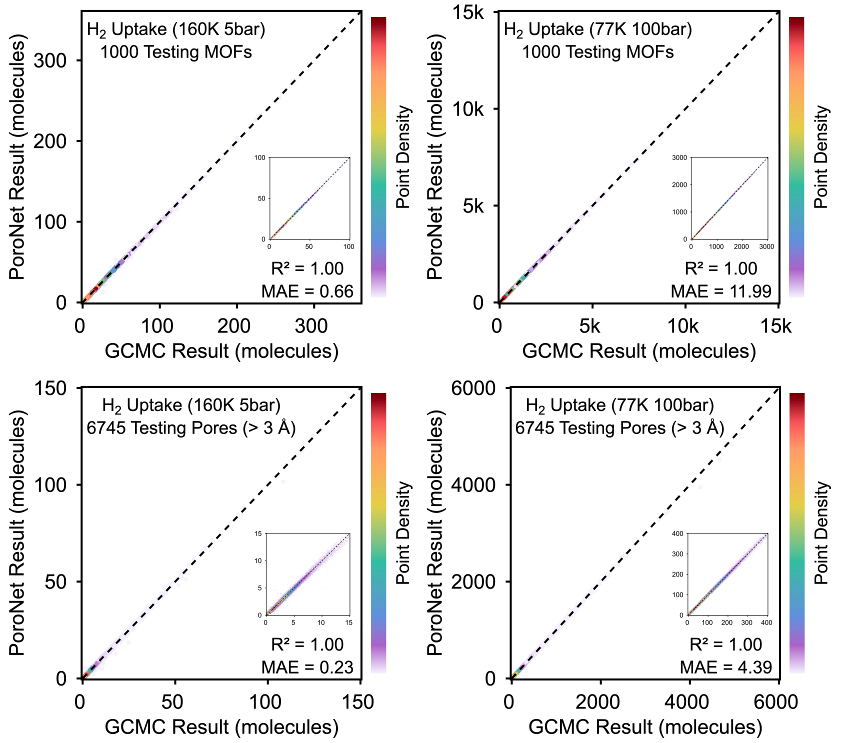

Figure S5. Parity plots comparing GCMC adsorption molecule number for H_2 adsorption (160 K/5 bar and 77 K/100 bar) at both MOF level and pore level, against PoroNet predictions on the testing set (1000 testing MOFs, 6745 testing pores). PoroNet models were trained on both pore-level and MOF-level labels.

S4. PoroNet-Base Prediction

S4.1 Parity Plots on Training Data for H_2 Adsorption under Cryogenic Conditions

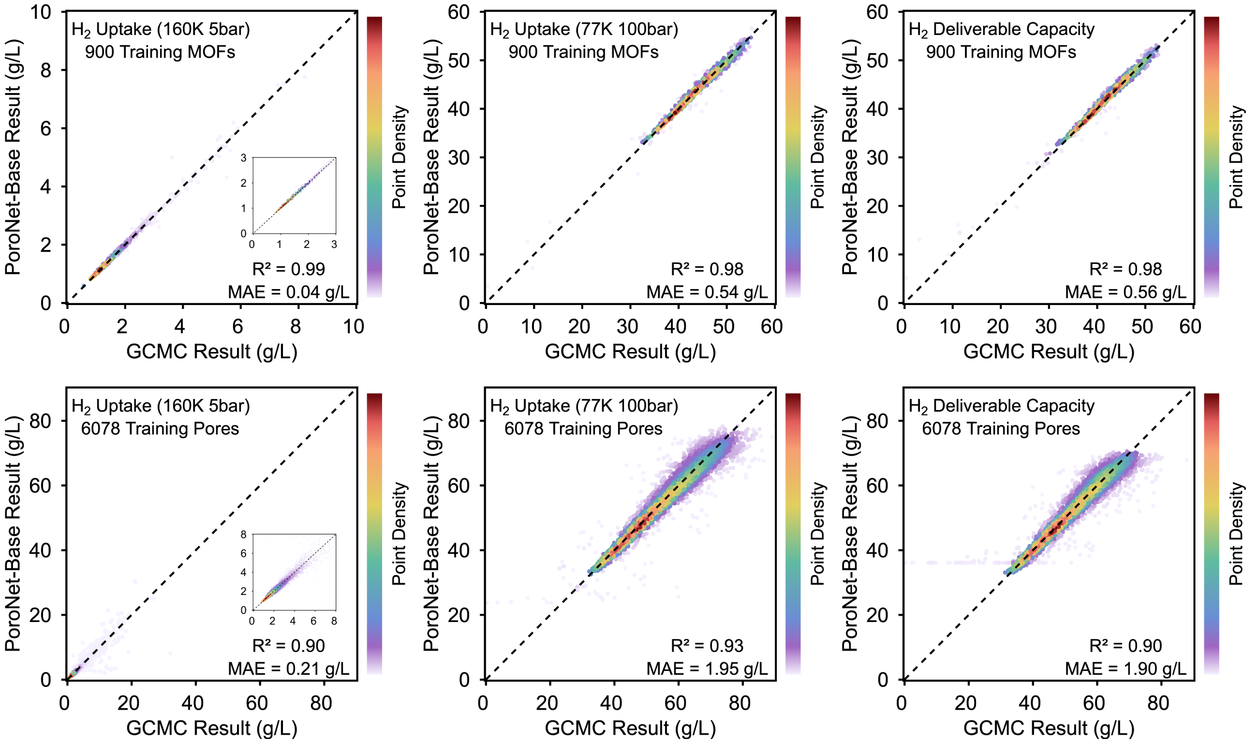

Figure S6. Parity plots comparing GCMC adsorption capacity for H_2 adsorption (160 K/5 bar and 77 K/100 bar) and deliverable capacity at both MOF level and pore level, against PoroNet-Base predictions on the training set (900 training MOFs, 6078 training pores). PoroNet-Base models were trained solely on MOF-level labels.

S4.2 Parity Plots on Testing Data for H_2 Adsorption at Room Temperature

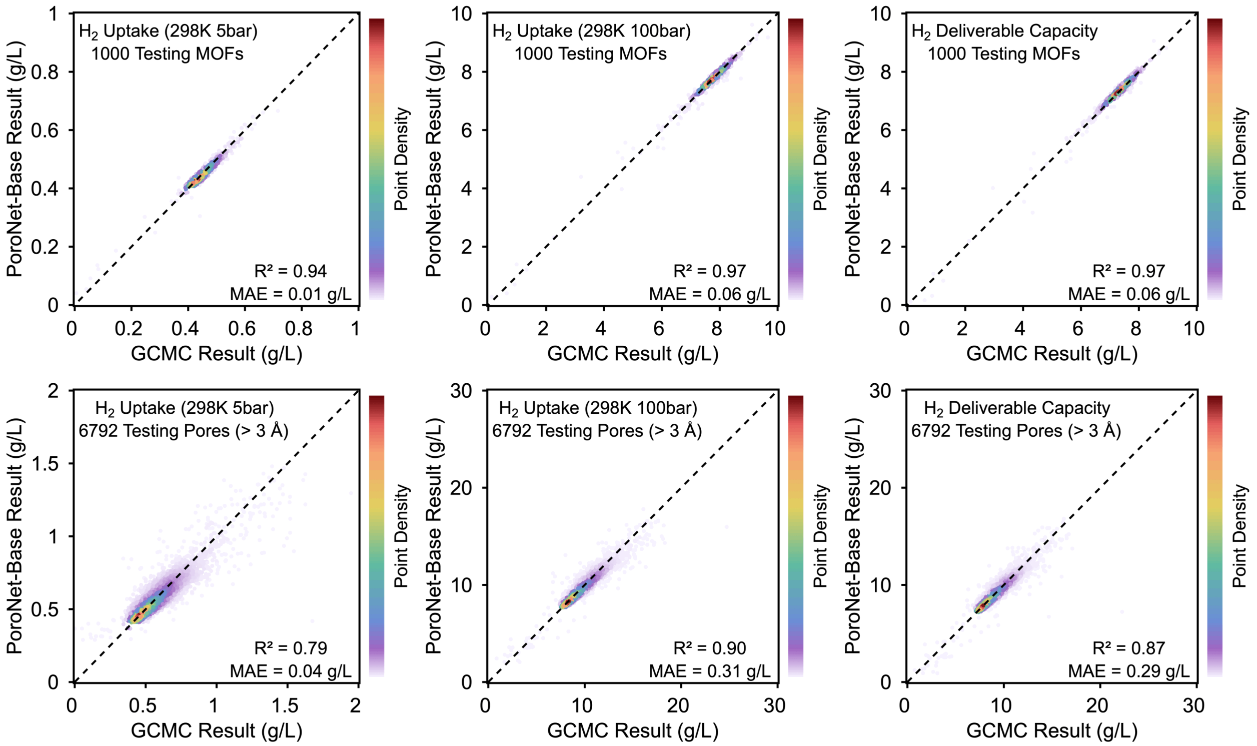

Figure S7. Parity plots comparing GCMC adsorption capacity for H_2 adsorption (298 K/5 bar and 298 K/100 bar) and deliverable capacity at both MOF level and pore level, against PoroNet-Base predictions on the testing set (1000 testing MOFs, 6792 testing pores). PoroNet-Base models were trained solely on MOF-level labels.

S4.3 Parity Plots on Testing Data for Predicted Adsorbed H_2 Molecule Numbers

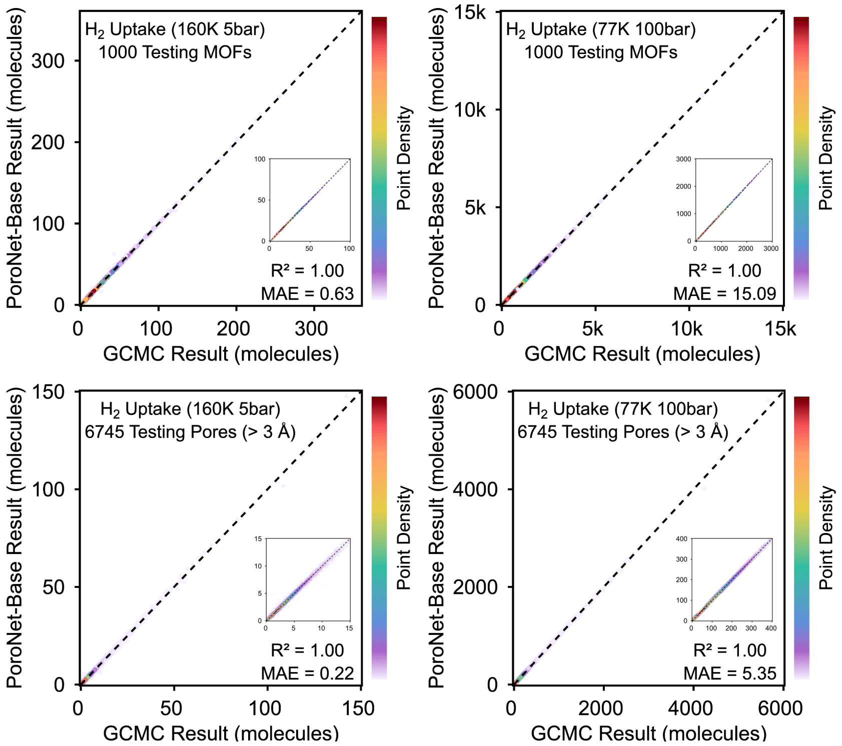

Figure S8. Parity plots comparing GCMC adsorption number for H_2 adsorption (160 K/5 bar and 77 K/100 bar) at both MOF level and pore level, against PoroNet-Base predictions on the testing set (1000 testing MOFs, 6745 testing pores). PoroNet-Base models were trained solely on MOF-level labels.

S4.4 Parity Plots on Testing Data for the Prediction of Adsorption of Other Gas Molecules in MOFs

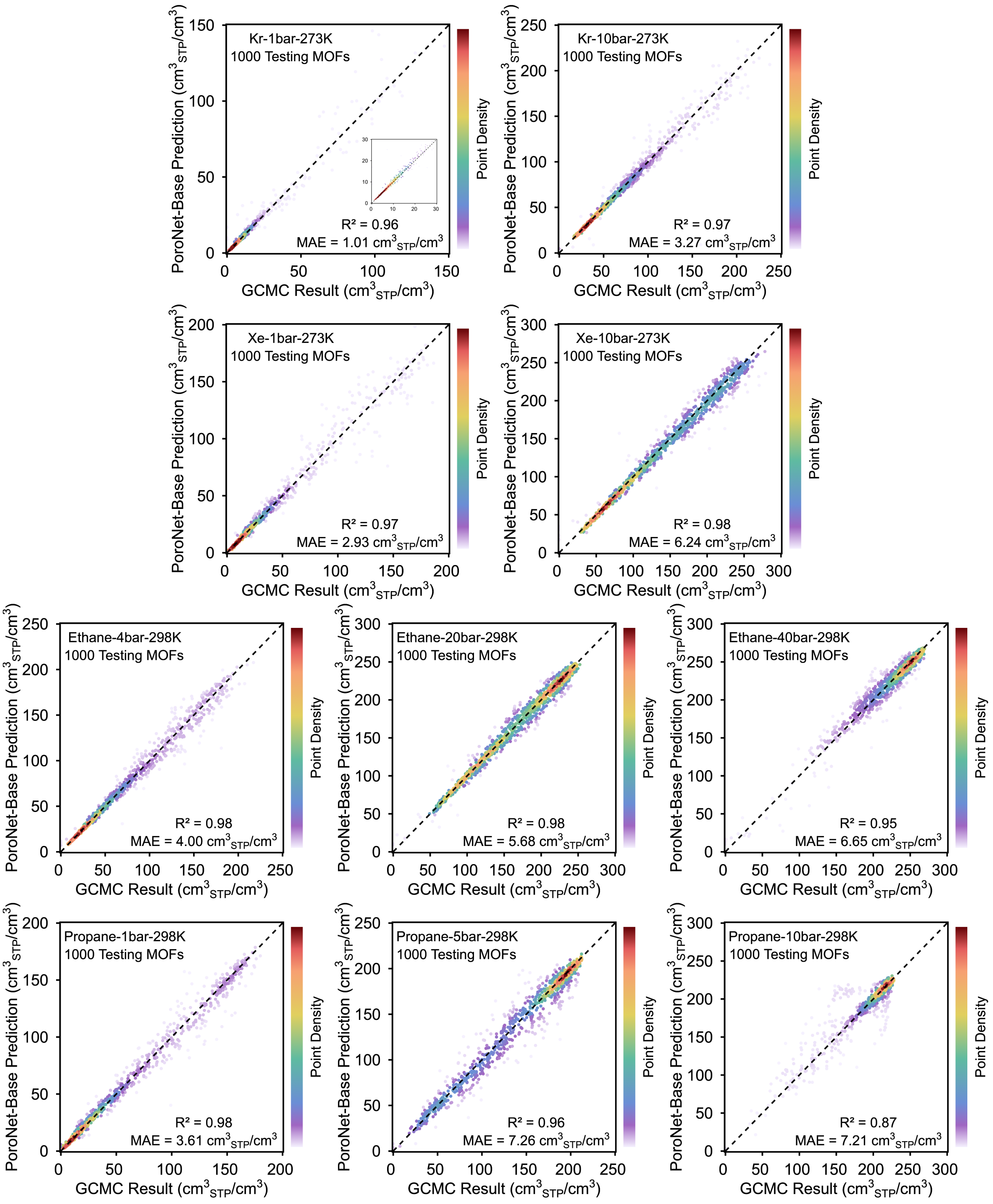

Figure S9. Parity plots comparing GCMC adsorption capacity for adsorption of Kr, Xe, ethane, and propane at the MOF level, against PoroNet-Base predictions on the testing set (1000 testing MOFs). PoroNet-Base models were trained solely on MOF-level labels.

S5. Data Efficiency of ML Models

S5.1 Learning Curves for Predicted H_2 Deliverable Capacity

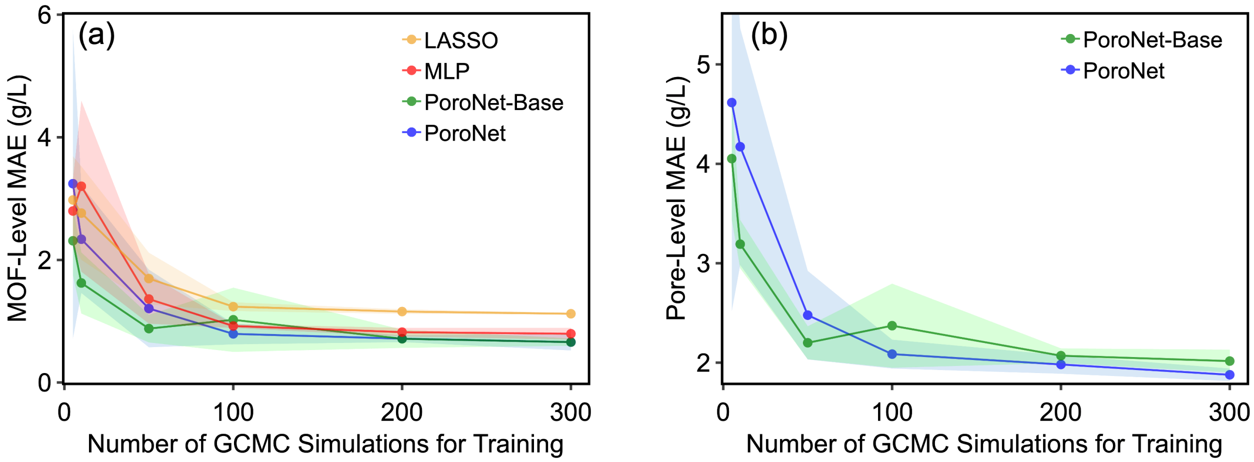

Figure S10. Data efficiency of ML models for predicting volumetric H_2 deliverable capacity at cryogenic conditions at the (a) MOF level and (b) pore level. The same testing samples as the density prediction in Secs. 3.1 and 3.2 were used to compute MAEs. Each point reports an average of testing MAEs from five independent runs with random splits of training samples, and the shaded area represents the corresponding standard deviation. The hyperparameters were re-optimized independently for each of the five runs underlying every point.

S6. High-Throughput Screening of Pores

S6.1 UMAP Analysis

Uniform Manifold Approximation and Projection (UMAP)[11] method for dimensionality reduction of pore-level energy histogram or cavity size distribution features was performed using the umap-learn package (version 0.5.7). The hyperparameters were set to: *n_neighbors* = 200, *min_dist* = 0.2, with a random seed = 5.

The code to calculate pore-level cavity size distribution is available at https://github.com/Shi-Research-group/PoroNet/tree/main/Cavity_Size_Distribution_Calculation.

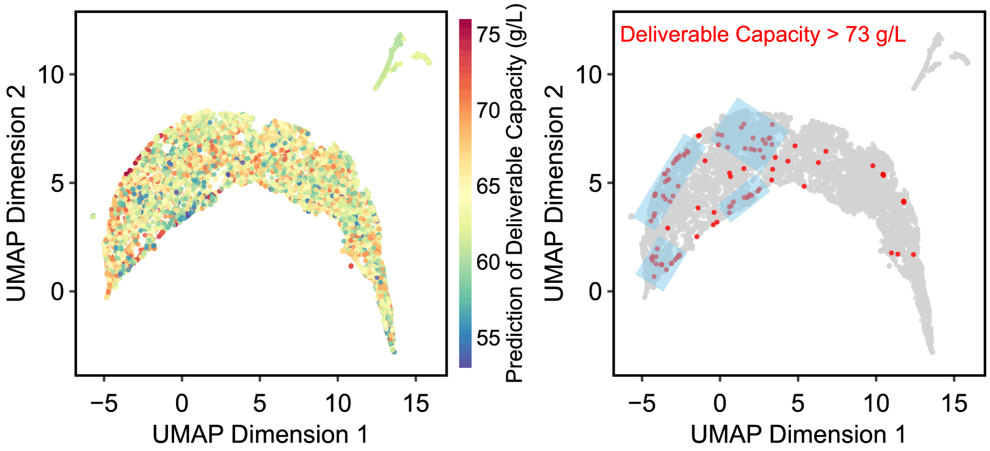

Figure S11. UMAP analysis of 10,953 pores in the Topologically Based Crystal Constructor (ToBaCCo) MOFs with a diameter from 8 to 11 Å. Cavity size distributions are used as input for dimensionality reduction. Each point represents a pore projected in a two-dimensional UMAP space, with points colored according to the PoroNet-predicted H_2 deliverable capacity at cryogenic conditions. The right figure highlights pores with deliverable capacity larger than 73 g/L, which appear to have clustered distributions, suggesting similarities in their shapes that favor H_2 storage.

S6.2 Applying PoroNet to Explain Adsorption in Experimental Nanoporous Materials

Table S9. Comparison of reported experimental and simulated data (from previous studies[2, 12-15]) against PoroNet predictions for MOF-level H_2 deliverable capacities at cryogenic conditions for selected framework materials from literature.

Material	Experiment (g/L)	Simulation (g/L)	PoroNet (g/L)
RP-H101[12]	53.70	46.80	46.79
MOF-5[13]	51.90	N.A.	49.99
HKUST-1[14]	46.00	48.00	46.54
PCN-61[2]	47.90	51.80	48.69
ntt_92[2]	N.A.	50.90	51.37
MFU-4l[15]	47.00	54.00	49.86

S6.3 SHapley Additive exPlanation (SHAP) Analysis

In addition to the inherent *interpretability* in the PoroNet architecture, we also show the *explainability* of PoroNet in the prediction of pore-level H_2 deliverable capacities at cryogenic conditions on the testing dataset using the SHAP package (version 0.48.0). The SHAP quantifies each feature's contribution to the output of PoroNet by calculating SHAP values. A positive (or negative) SHAP value represents a positive (or negative) contribution to the ML prediction of pore-level deliverable capacity. As shown in the SHAP summary plot (Figure S12a), points representing the pores in the testing set are colored with the energy histogram feature values. In the energy range of (−1, +∞), the higher the positive SHAP values are, the lower the feature value is, which indicates a negative effect of this energy range on cryogenic H_2 deliverable capacity. This is because the higher ratio of repulsive region (E ≥ 0 kJ/mol) represents a larger portion of pore space that is exclusive to H_2 adsorption. In contrast, the energy range of (−7, −1] has a positive impact on the H_2 deliverable capacity due to the optimal attractive interactions between H_2 molecules and MOF frameworks. Nevertheless, energies below −7 kJ/mol appear to negatively influence the deliverable capacity, because attractive interactions that are too strong make it harder for H_2 molecules to desorb at 160 K/5 bar.[16] A similar pattern was observed in a previous study, where adsorption energies below −6 kJ/mol were identified to have a negative impact on the cryogenic H_2 deliverable capacity via the least absolute shrinkage and selection operator (LASSO) model.[15] Figure S12b illustrates the mean absolute SHAP value for each feature, reflecting the overall influence of different energy bins on deliverable capacity. The energy bins (−7, −1] have mean absolute SHAP values of 0.37–4.19, showing significant overall contributions. Among energy bins with a negative impact, (0, +∞) and (−1, 0] exhibit significantly higher mean absolute SHAP values (2.33 and 1.82) compared to other bins in the range of (−∞, −7] (0.03–0.06), indicating their dominant unfavorable influence. Therefore, a higher cryogenic H_2 deliverable capacity in a pore results from a greater fraction of spaces with energies in the favorable range (−7, −1], coupled with a smaller fraction of space with unfavorable energies of (−1, +∞) and (−∞, −7].

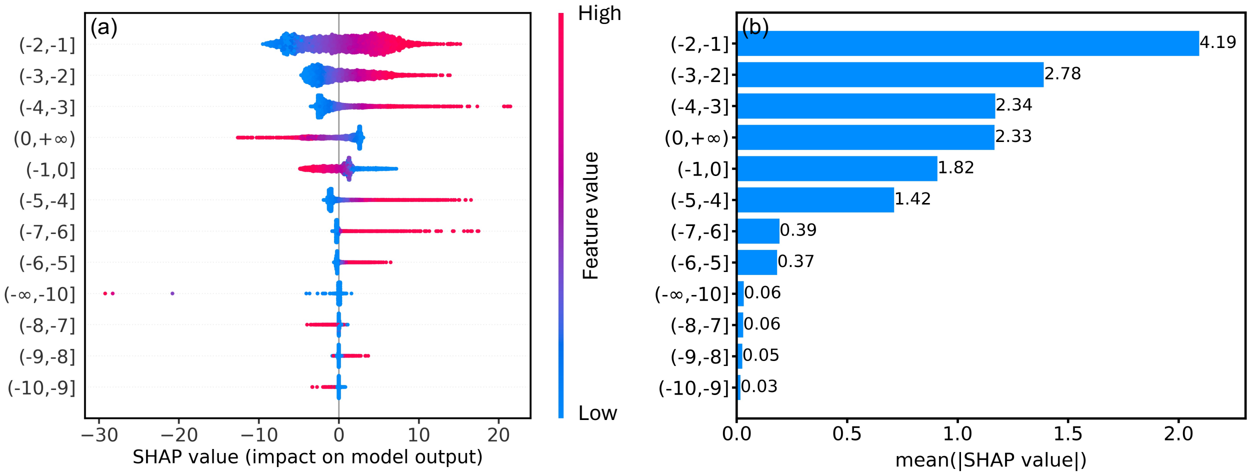

Figure S12. (a) SHAP summary plot and (b) mean absolute SHAP values for different bins in the pore-level energy histogram.

S6.4 Visualization of the Significant Outlier

A significant outlier pore was observed in the original analysis (Figure S13). We found that it is an unphysical pore consisting of two lines of energy grid points (Table S10), which can be attributed to the imperfect pore segmentation algorithm we used. However, this limitation does not compromise the reliability of our work or the validity of the data analysis presented in this study, as such unphysically segmented pores are rare.

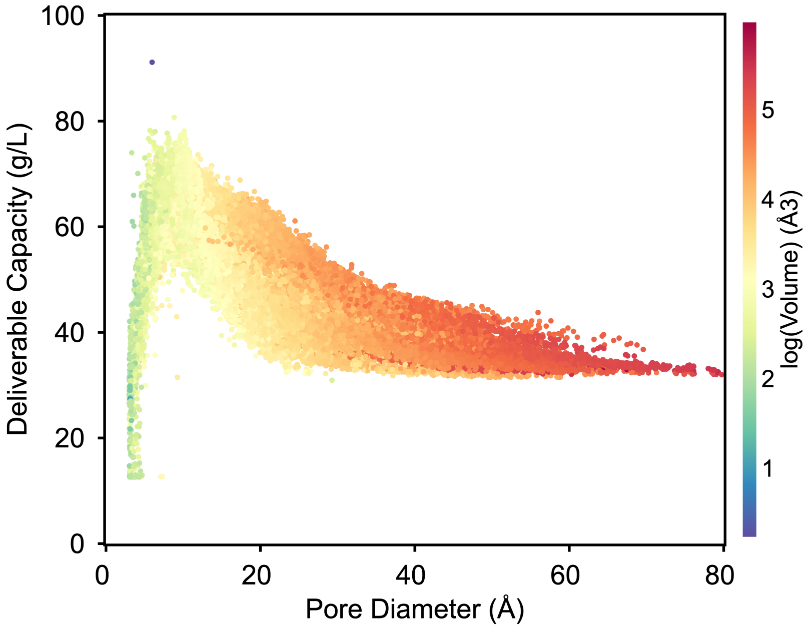

Figure S13. The original distribution of predicted pore-level H_2 deliverable capacities at cryogenic conditions by PoroNet as a function of the pore diameter for a total of 89,773 accessible pores in the ToBaCCo database. There is an extreme outlier at 5.97 Å (deliverable capacity over 90 g/L), which has an extremely small volume. This figure corresponds to Figure 6 in the main text.

Table S10. Structural information of the outlier pore in Figure S13.

Structure	Diameter (Å)	Pore-Level Deliverable Capacity at Cryogenic Conditions (g/L)	MOF	Pore Index
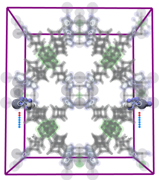	5.97	91.15	tobmof-5964	2

S6.5 Effects of Pore-Level Surface Area and Chemistry on Cryogenic H_2 Deliverable Capacity

Using both the LASSO model and GCMC simulations, Bucior et al.[15] illustrated that the metal elements of MOFs do not have a significant effect on cryogenic H_2 deliverable capacities. Our tests also showed similar results. Moreover, we selected pores with a diameter within 8.4–8.7 Å and analyzed the relationship between cryogenic H_2 deliverable capacity and pore-level VSA in units of m^2/cm^3(pore). The definition of pore-level VSA is listed in Table S5. As shown in Figure S14, H_2 deliverable capacity appears to be independent of pore-level VSA.

The code to calculate the pore-level VSA is available at https://github.com/Shi-Research-Group/PoroNet/tree/main/Pore_Surface_Area_Calculation.

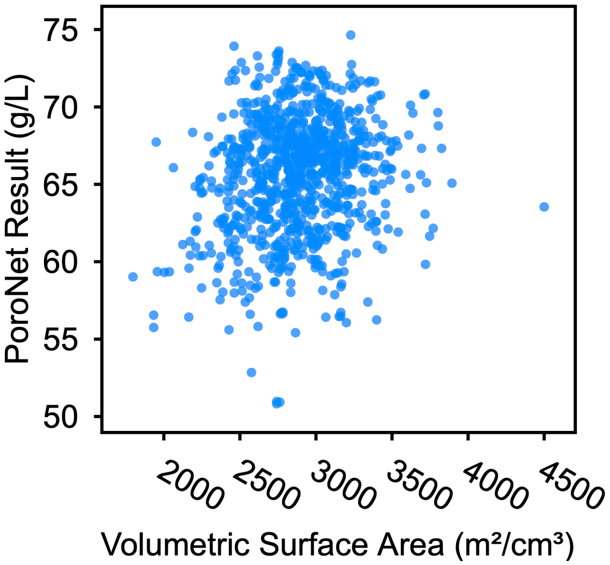

Figure S14. Distribution of predicted H_2 deliverable capacities at cryogenic conditions as a function of pore-level VSA. Each point represents a pore with a diameter in the range of 8.4–8.7 Å in ToBaCCo MOFs.

S6.6 Visualization of Top-Performing Pores

Table S11. A list of top-performing pores with a diameter ranging from 8 to 11 Å. The color scheme of the pore region is the same as that in Figure 6 in the main text.

Pore	Structure	Pore-Level Deliverable Capacity at Cryogenic Conditions (g/L)	Complete/Partial	Pore Shape	MOF	Pore Index in the Pore Graph
1	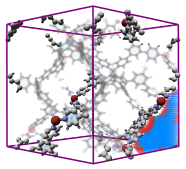	80.73	Partial	N.A.	tobmof-1792	2
2	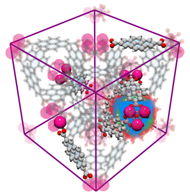	78.14	Complete	Triangular pyramid	tobmof-2369	10
3	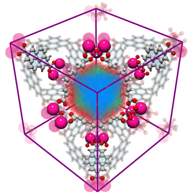	77.58	Complete	Triangular pyramid	tobmof-2369	9
4	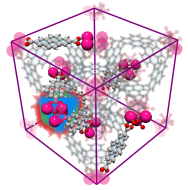	77.44	Complete	Triangular pyramid	tobmof-2369	2

5	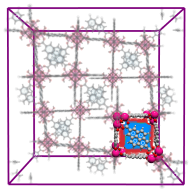	77.40	Complete	Triangular pyramid	tobmof-817	9
6	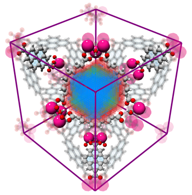	77.13	Complete	Triangular pyramid	tobmof-2369	5
7	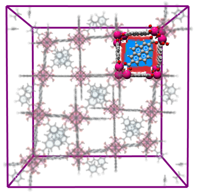	77.10	Complete	Triangular pyramid	tobmof-817	4
8	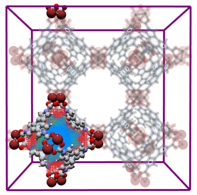	76.90	Complete	Square bipyramid	tobmof-12354	7
9	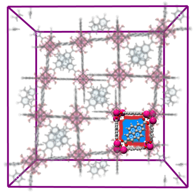	76.68	Complete	Triangular pyramid	tobmof-817	3

10	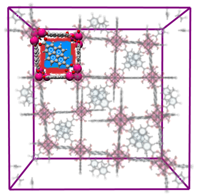	76.64	Complete	Triangular pyramid	tobmof-817	8
11	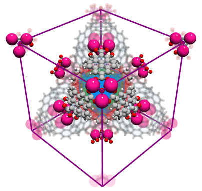	76.59	Complete	Triangular pyramid	tobmof-2369	3
12	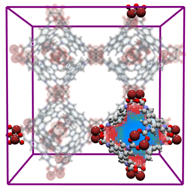	76.50	Complete	Square bipyramid	tobmof-12354	14
13	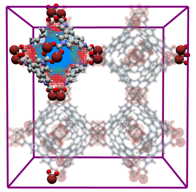	76.46	Complete	Square bipyramid	tobmof-12354	4
14	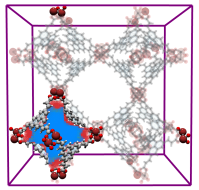	76.44	Complete	Square bipyramid	tobmof-12328	4

15	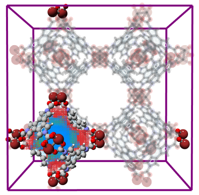	76.42	Complete	Square bipyramid	tobmof-12354	13
16	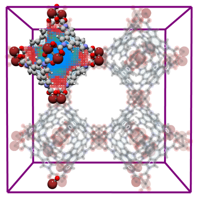	76.36	Complete	Square bipyramid	tobmof-12354	12
17	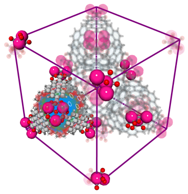	76.35	Complete	Triangular pyramid	tobmof-2369	8
18	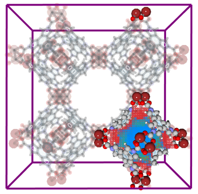	76.32	Complete	Square bipyramid	tobmof-12354	5
19	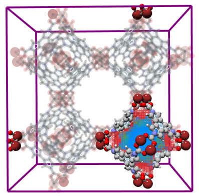	76.29	Complete	Square bipyramid	tobmof-12354	6

20	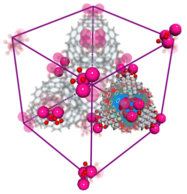	76.23	Complete	Triangular pyramid	tobmof-2369	4
21	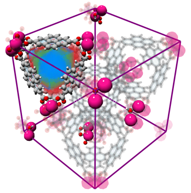	75.89	Complete	Triangular pyramid	tobmof-2369	11
22	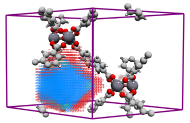	75.87	Partial	N.A.	tobmof-8	2
23	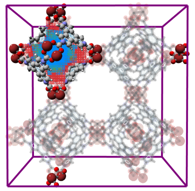	75.83	Complete	Square bipyramid	tobmof-12354	15
24	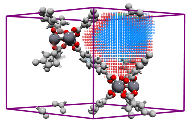	75.74	Partial	N.A.	tobmof-8	2

25	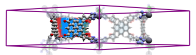	75.71	Partial	N.A.	tobmof-1145	1
26	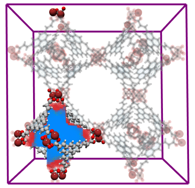	75.68	Complete	Square bipyramid	tobmof-12328	12
27	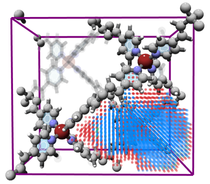	75.50	Partial	N.A.	tobmof-2048	1
28	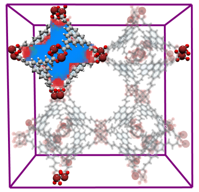	75.37	Complete	Square bipyramid	tobmof-12328	13
29	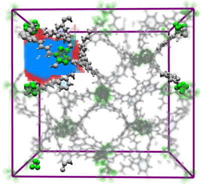	75.33	Partial	N.A.	tobmof-3735	5

30	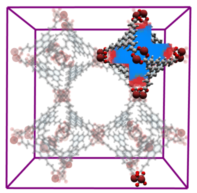	75.23	Complete	Square bipyramid	tobmof-12328	5
31	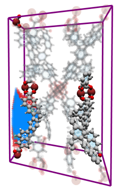	75.20	Partial	N.A.	tobmof-11312	4
32	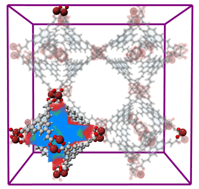	75.15	Complete	Square bipyramid	tobmof-12328	14
33	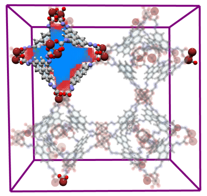	75.05	Complete	Square bipyramid	tobmof-12317	12
34	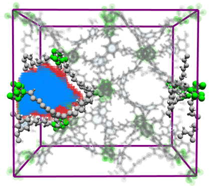	74.99	Partial	N.A.	tobmof-3735	13

35	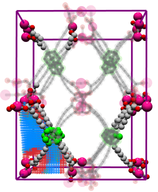	74.95	Partial	N.A.	tobmof-5878	2
36	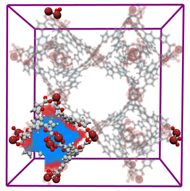	74.88	Complete	Square bipyramid	tobmof-12301	12
37	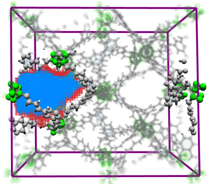	74.70	Partial	N.A.	tobmof-3734	13
38	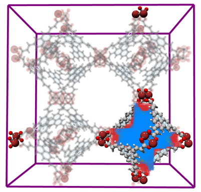	74.67	Complete	Square bipyramid	tobmof-12328	7
39	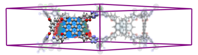	74.64	Partial	N.A.	tobmof-1145	4

40	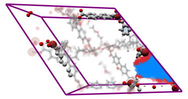	74.50	Partial	N.A.	tobmof-5164	3
41	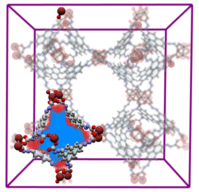	74.44	Complete	Square bipyramid	tobmof-12317	7
42	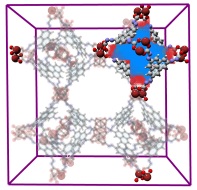	74.35	Complete	Square bipyramid	tobmof-12317	4
43	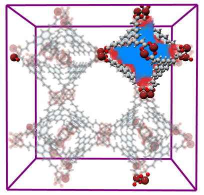	74.28	Complete	Square bipyramid	tobmof-12328	6
44	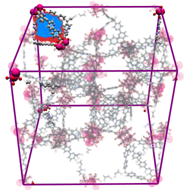	74.22	Complete	Triangular bipyramid	tobmof-3842	27

45	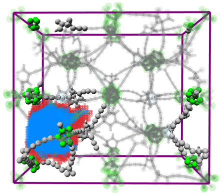	74.14	Partial	N.A.	tobmof-3640	10
46	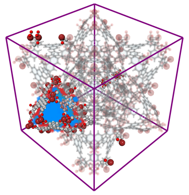	74.10	Complete	Square bipyramid	tobmof-8718	12
47	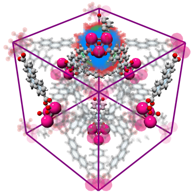	74.08	Complete	Triangular pyramid	tobmof-2370	8
48	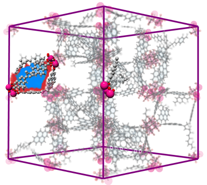	74.05	Complete	Triangular bipyramid	tobmof-3887	25
49	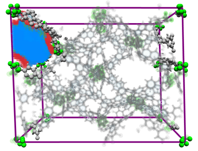	74.04	Partial	N.A.	tobmof-3737	11

50	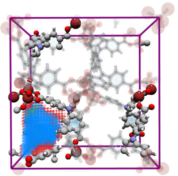	74.04	Partial	N.A.	tobmof-7589	4
51	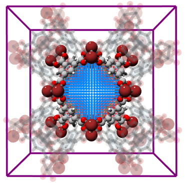	74.04	Complete	Truncated square bipyramid	tobmof-12258	11
52	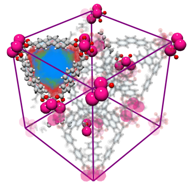	74.03	Complete	Triangular pyramid	tobmof-2370	3
53	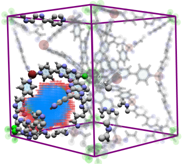	74.00	Complete	Triangular bipyramid	tobmof-3978	3
54	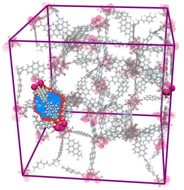	73.94	Complete	Triangular bipyramid	tobmof-3887	19

55	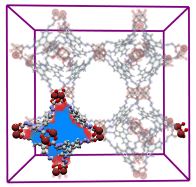	73.93	Complete	Square bipyramid	tobmof-12365	6
56	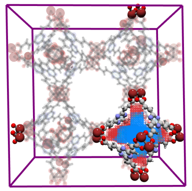	73.92	Complete	Square bipyramid	tobmof-12232	12
57	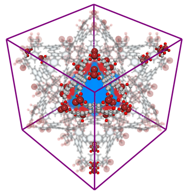	73.89	Complete	Square bipyramid	tobmof-8718	15
58	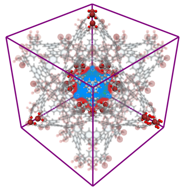	73.86	Complete	Square bipyramid	tobmof-8718	4
59	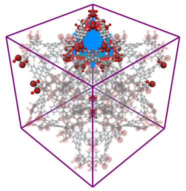	73.81	Complete	Square bipyramid	tobmof-8718	5

60	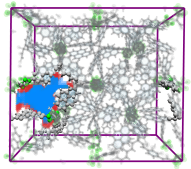	73.80	Partial	N.A.	tobmof-3551	7
61	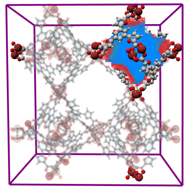	73.80	Complete	Square bipyramid	tobmof-12301	4
62	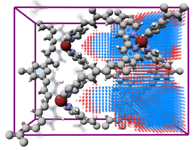	73.78	Partial	N.A.	tobmof-2122	1
63	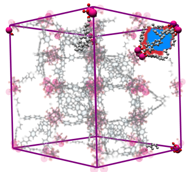	73.75	Complete	Triangular bipyramid	tobmof-3841	25
64	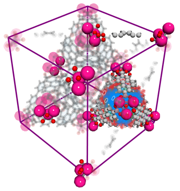	73.70	Complete	Triangular pyramid	tobmof-2370	4

65	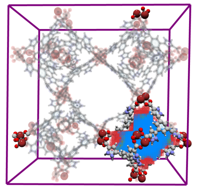	73.70	Complete	Square bipyramid	tobmof-12300	11
66	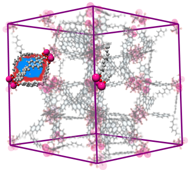	73.69	Complete	Triangular bipyramid	tobmof-3840	18
67	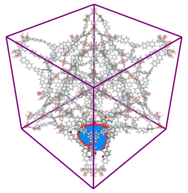	73.62	Complete	Triangular pyramid	tobmof-8731	29
68	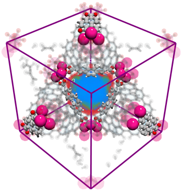	73.62	Complete	Triangular pyramid	tobmof-2370	9
69	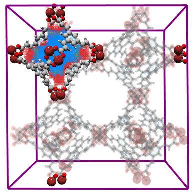	73.62	Complete	Square bipyramid	tobmof-12280	12

70	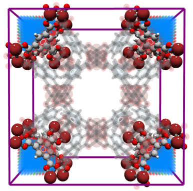	73.61	Complete	Truncated square bipyramid	tobmof-12258	8
71	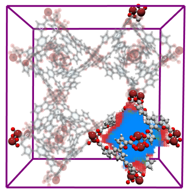	73.59	Complete	Square bipyramid	tobmof-12301	13
72	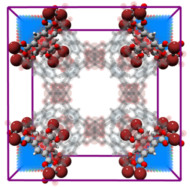	73.58	Complete	Truncated square bipyramid	tobmof-12258	2
73	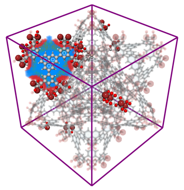	73.57	Complete	Square bipyramid	tobmof-8718	13
74	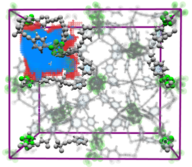	73.57	Partial	N.A.	tobmof-3549	4

75	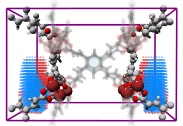	73.54	Partial	N.A.	tobmof-11566	1
76	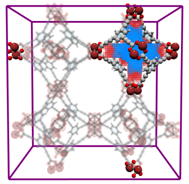	73.53	Complete	Square bipyramid	tobmof-12291	15
77	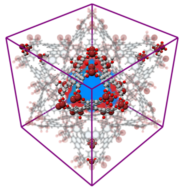	73.53	Complete	Square bipyramid	tobmof-8718	7
78	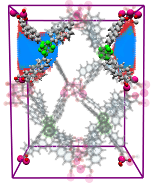	73.50	Complete	Square bipyramid-like	tobmof-5891	7
79	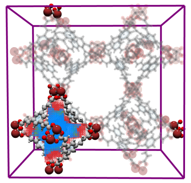	73.49	Complete	Square bipyramid	tobmof-12280	5

80	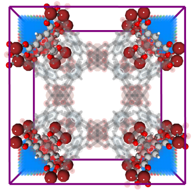	73.48	Complete	Truncated square bipyramid	tobmof-12258	1
81	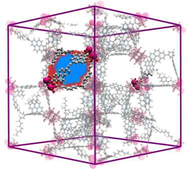	73.48	Complete	Triangular bipyramid	tobmof-3887	2
82	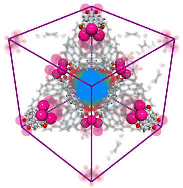	73.48	Complete	Triangular pyramid	tobmof-2370	2
83	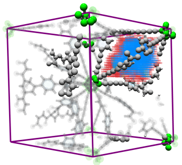	73.48	Complete	Triangular bipyramid	tobmof-4137	11
84	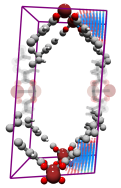	73.46	Partial	N.A.	tobmof-7159	0

85	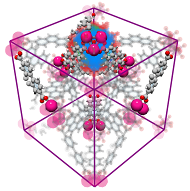	73.43	Complete	Triangular pyramid	tobmof-2370	5
86	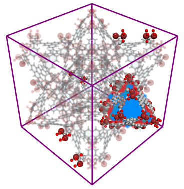	73.39	Complete	Square bipyramid	tobmof-8718	6
87	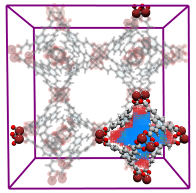	73.38	Complete	Square bipyramid	tobmof-12280	14
88	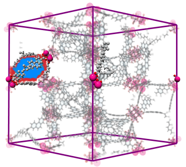	73.35	Complete	Triangular bipyramid	tobmof-3888	25
89	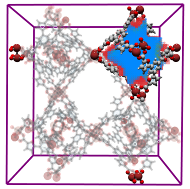	73.34	Complete	Square bipyramid	tobmof-12301	11

90	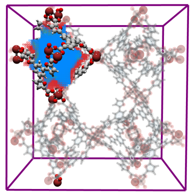	73.33	Complete	Square bipyramid	tobmof-12301	5
91	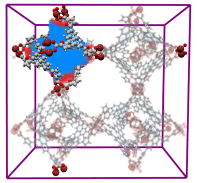	73.33	Complete	Square bipyramid	tobmof-12349	11
92	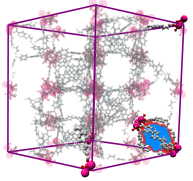	73.32	Complete	Triangular bipyramid	tobmof-3841	2
93	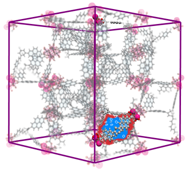	73.31	Complete	Triangular bipyramid	tobmof-3887	13
94	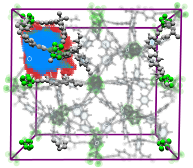	73.30	Partial	N.A.	tobmof-3547	2

95	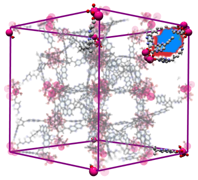	73.30	Complete	Triangular bipyramid	tobmof-3882	20
96	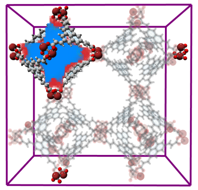	73.29	Complete	Square bipyramid	tobmof-12328	15
97	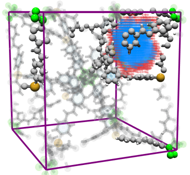	73.29	Complete	Triangular bipyramid	tobmof-4044	6
98	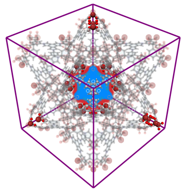	73.28	Complete	Square bipyramid	tobmof-8766	4
99	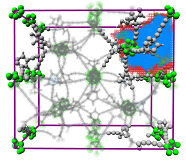	73.26	Partial	N.A.	tobmof-3640	0

100	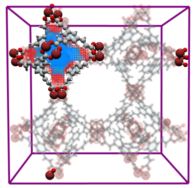	73.25	Complete	Square bipyramid	tobmof-12280	7
101	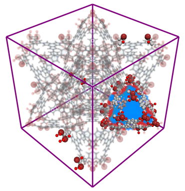	73.19	Complete	Square bipyramid	tobmof-8766	7
102	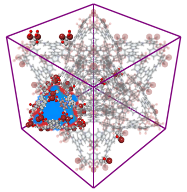	73.19	Complete	Square bipyramid	tobmof-8766	14
103	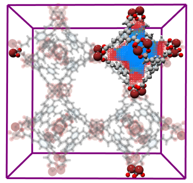	73.18	Complete	Square bipyramid	tobmof-12280	6
104	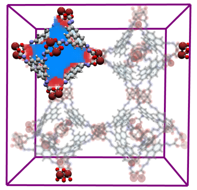	73.18	Complete	Square bipyramid	tobmof-12317	5

105	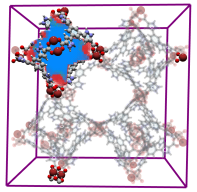	73.18	Complete	Square bipyramid	tobmof-12300	6
106	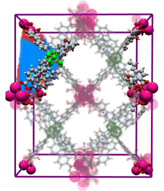	73.17	Partial	N.A.	tobmof-5980	2
107	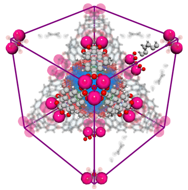	73.17	Complete	Triangular pyramid	tobmof-2370	10
108	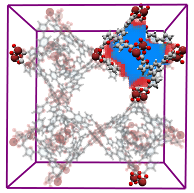	73.16	Complete	Square bipyramid	tobmof-12301	14
109	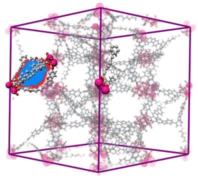	73.14	Complete	Triangular bipyramid	tobmof-3886	25

110	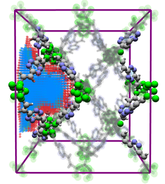	73.13	Partial	N.A.	tobmof-117	3
111	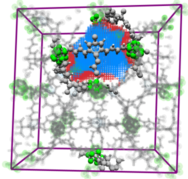	73.12	Complete	Irregular	tobmof-3639	12
112	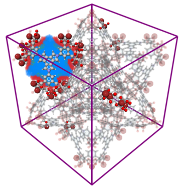	73.12	Complete	Square bipyramid	tobmof-8766	12
113	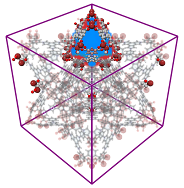	73.12	Complete	Square bipyramid	tobmof-8766	6
114	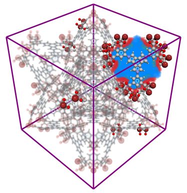	73.10	Complete	Square bipyramid	tobmof-8766	5

115	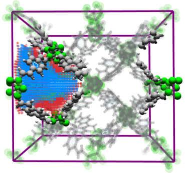	73.09	Partial	N.A.	tobmof-115	0
116	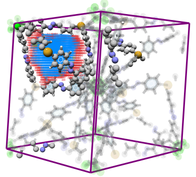	73.08	Complete	Triangular bipyramid	tobmof-4046	6
117	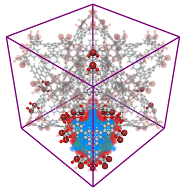	73.08	Complete	Square bipyramid	tobmof-8718	14
118	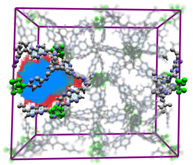	73.03	Partial	N.A.	tobmof-3642	8
119	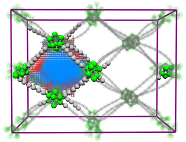	73.03	Complete	Square bipyramid-like	tobmof-113	6

120	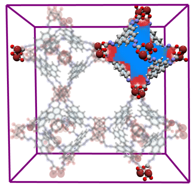	73.03	Complete	Square bipyramid	tobmof-12317	14
121	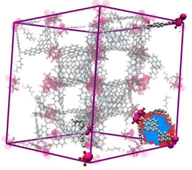	73.00	Complete	Triangular bipyramid	tobmof-3839	3

S7. Methods Benchmark

Table S12. Computational time and storage comparison between typical post-analysis and PoroNet-Base approaches. All data reported are measured from this study.

	Post-Analysis of Simulation Trajectories	PoroNet-Base
Average GCMC Simulation Time per MOF (160 K)	0.3 CPU hours	0.3 CPU hours
Required MOF Number	13,511	1,000
GCMC Simulation Time (160 K)	~4,053 CPU hours	300 CPU hours
Average Storage per PDB File (160 K)	3 MB	3 MB
Required PDB Number	13,511	N.A.
Storage for PDB Files (160 K)	~40 GB	N.A.
Average GCMC Simulation Time per MOF (77 K)	40 CPU hours	40 CPU hours
Required MOF Number	13,511	1,000
GCMC Simulation Time (77 K)	540,440 CPU hours	40,000 CPU hours
Average Storage for per PDB File (77K)	110 MB	110 MB
Required PDB Number	13,511	N.A.
Storage for PDB Files (77 K)	~1,451 GB	N.A.
Average Pore Graph Generation Time	200 CPU seconds	200 CPU seconds
Required Pore Graph Number	N.A.	13,511
Pore Graph Generation Time	N.A.	~751 CPU hours
Average Storage per Pore Graph	15 KB	15 KB
Required Pore Graph Number	N.A.	13,511
Storage for Pore Graphs	N.A.	~198 MB
Model Training Time	N.A.	~60 CPU hours
Average Time for Pore-level Capacity per Pore (160 K)	25 CPU seconds	Negligible
Required Pore Number	90,411	90,411
Time for Pore-level Capacity (160 K)	~628 CPU hours	< 1 min

Average Time for Pore-level Capacity per Pore (77 K)<br>Required Pore Number<br>Time for Pore-level Capacity (77 K)	30 CPU seconds<br>90,114<br>~753 CPU hours	Negligible<br>90,114<br>< 1 min
Total Time	~545,874 CPU hours	~41,111 CPU hours
Total Storage	~1,491 GB (~1.5 TB)	~198 MB

References

(1) Darkrim, F.; Levesque, D. Monte Carlo simulations of hydrogen adsorption in single-walled carbon nanotubes. *The Journal of Chemical Physics* **1998**, *109* (12), 4981-4984.
(2) Liu, K.; Chen, Z.; Islamoglu, T.; Lee, S.-J.; Chen, H.; Yildirim, T.; Farha, O. K.; Snurr, R. Q. Exploring the Chemical Space of Metal–Organic Frameworks with rht Topology for High Capacity Hydrogen Storage. *The Journal of Physical Chemistry C* **2024**, *128* (18), 7435-7446.
(3) Sun, Y. Z. S.; DeJaco, R. F.; Li, Z.; Tang, D.; Glante, S.; Sholl, D. S.; Colina, C. M.; Snurr, R. Q.; Thommes, M.; Hartmann, M.; et al. Fingerprinting diverse nanoporous materials for optimal hydrogen storage conditions using meta-learning. *Sci. Adv.* **2021**, *7* (30), 12.
(4) Dubbeldam, D.; Calero, S.; Ellis, D. E.; Snurr, R. Q. RASPA: molecular simulation software for adsorption and diffusion in flexible nanoporous materials. *Molecular Simulation* **2015**, *42* (2), 81-101.
(5) Bondi, A. van der Waals Volumes and Radii. *The Journal of Physical Chemistry* **1964**, *68* (3), 441-451.
(6) Soille, P. J.; Ansoult, M. M. AUTOMATED BASIN DELINEATION FROM DIGITAL ELEVATION MODELS USING MATHEMATICAL MORPHOLOGY. *Signal Process.* **1990**, *20* (2), 171-182.
(7) Paszke, A.; Gross, S.; Massa, F.; Lerer, A.; Bradbury, J.; Chanan, G.; Killeen, T.; Lin, Z. M.; Gimelshein, N.; Antiga, L.; et al. PyTorch: An Imperative Style, High-Performance Deep Learning Library. In *33rd Conference on Neural Information Processing Systems (NeurIPS)*, Vancouver, CANADA, Dec 08-14, 2019; Neural Information Processing Systems (Nips): LA JOLLA, 2019; Vol. 32.
(8) Wang, M.; Zheng, D.; Ye, Z.; Gan, Q.; Li, M.; Song, X.; Zhou, J.; Ma, C.; Yu, L.; Gai, Y. Deep graph library: A graph-centric, highly-performant package for graph neural networks. *arXiv preprint arXiv:1909.01315* **2019**.
(9) Akiba, T.; Sano, S.; Yanase, T.; Ohta, T.; Koyama, M. Optuna: A Next-generation Hyperparameter Optimization Framework. In Proceedings of the 25th ACM SIGKDD International Conference on Knowledge Discovery & Data Mining, 2019.
(10) Shi, K.; Li, Z.; Anstine, D. M.; Tang, D.; Colina, C. M.; Sholl, D. S.; Siepmann, J. I.; Snurr, R. Q. Two-Dimensional Energy Histograms as Features for Machine Learning to Predict Adsorption in Diverse Nanoporous Materials. *J. Chem. Theory Comput.* **2023**, *19* (14), 4568-4583.
(11) McInnes, L.; Healy, J.; Melville, J. UMAP: Uniform Manifold Approximation and Projection for Dimension Reduction. *arXiv* **2020**.
(12) Zhang, R.; Daglar, H.; Tang, C.; Li, P.; Feng, L.; Han, H.; Wu, G.; Limketkai, B. N.; Wu, Y.; Yang, S.; et al. Balancing volumetric and gravimetric capacity for hydrogen in supramolecular crystals. *Nat Chem* **2024**, *16* (12), 1982-1988.
(13) Ahmed, A.; Liu, Y.; Purewal, J.; Tran, L. D.; Wong-Foy, A. G.; Veenstra, M.; Matzger, A. J.; Siegel, D. J. Balancing gravimetric and volumetric hydrogen density in MOFs. *Energy Environ. Sci.* **2017**, *10* (11), 2459-2471.
(14) García-Holley, P.; Schweitzer, B.; Islamoglu, T.; Liu, Y.; Lin, L.; Rodriguez, S.; Weston, M. H.; Hupp, J. T.; Gómez-Gualdrón, D. A.; Yildirim, T.; et al. Benchmark Study of Hydrogen Storage in Metal–Organic Frameworks under Temperature and Pressure Swing Conditions. *ACS Energy Letters* **2018**, *3* (3), 748-754.

(15) Bucior, B. J.; Bobbitt, N. S.; Islamoglu, T.; Goswami, S.; Gopalan, A.; Yildirim, T.; Farha, O. K.; Bagheri, N.; Snurr, R. Q. Energy-Based Descriptors to Rapidly Predict Hydrogen Storage in Metal–Organic Frameworks. *Molecular Systems Design & Engineering* **2019**, *4* (1), 162-174.
(16) Bobbitt, N. S.; Chen, J. Y.; Snurr, R. Q. High-Throughput Screening of Metal-Organic Frameworks for Hydrogen Storage at Cryogenic Temperature. *J. Phys. Chem. C* **2016**, *120* (48), 27328-27341.
